# Moonlighting of *Haemophilus influenzae* heme acquisition systems contributes to the host airway-pathogen interplay in a coordinated manner

**DOI:** 10.1080/21505594.2019.1596506

**Published:** 2019-04-11

**Authors:** Irene Rodríguez-Arce, Tamim Al-Jubair, Begoña Euba, Ariadna Fernández-Calvet, Celia Gil-Campillo, Sara Martí, Susanna Törnroth-Horsefield, Kristian Riesbeck, Junkal Garmendia

**Affiliations:** a Instituto de Agrobiotecnología, CSIC-Gobierno, Navarra, Spain; b Clinical Microbiology, Department of Translational Medicine, Faculty of Medicine, Lund University, Malmö, Sweden; c Department of Biomedical Sciences, Faculty of Health Sciences, University of Copenhagen, Copenhagen, Denmark; d Centro de Investigación Biomédica en Red de Enfermedades Respiratorias (CIBERES), Madrid, Spain; e Departamento Microbiología, Hospital Universitari Bellvitge, University of Barcelona, IDIBELL, Barcelona, Spain; f Department of Biochemistry and Structural Biology, Center for Molecular Protein Science, Lund University, Lund, Sweden

**Keywords:** *Haemophilus influenzae*, heme binding, iron nutritional immunity, protein moonlighting, respiratory infection

## Abstract

Nutrient iron sequestration is the most significant form of nutritional immunity and causes bacterial pathogens to evolve strategies of host iron scavenging. Cigarette smoking contains iron particulates altering lung and systemic iron homeostasis, which may enhance colonization in the lungs of patients suffering chronic obstructive pulmonary disease (COPD) by opportunistic pathogens such as nontypeable. NTHi is a heme auxotroph, and the NTHi genome contains multiple heme acquisition systems whose role in pulmonary infection requires a global understanding. In this study, we determined the relative contribution to NTHi airway infection of the four heme-acquisition systems HxuCBA, PE, SapABCDFZ, and HbpA-DppBCDF that are located at the bacterial outer membrane or the periplasm. Our computational studies provided plausible 3D models for HbpA, SapA, PE, and HxuA interactions with heme. Generation and characterization of single mutants in the *hxuCBA, hpe, sapA,* and *hbpA* genes provided evidence for participation in heme binding-storage and inter-bacterial donation. The *hxuA, sapA, hbpA,* and *hpe* genes showed differential expression and responded to heme. Moreover, HxuCBA, PE, SapABCDFZ, and HbpA-DppBCDF presented moonlighting properties related to resistance to antimicrobial peptides or glutathione import, together likely contributing to the NTHi-host airway interplay, as observed upon cultured airway epithelia and *in vivo* lung infection. The observed multi-functionality was shown to be system-specific, thus limiting redundancy. Together, we provide evidence for heme uptake systems as bacterial factors that act in a coordinated and multi-functional manner to subvert nutritional- and other sources of host innate immunity during NTHi airway infection.

## Introduction

The need of invading bacteria to acquire nutrient metals from their environments has caused vertebrate hosts to evolve to restrict their bioavailability. Nutrient iron sequestration is one mode of innate defence termed “iron nutritional immunity”, whereby iron levels are tightly controlled by host regulatory systems controlling its absorption, systemic transport, distribution, cellular uptake, and storage [1,2]. In response to this, pathogens evolve mechanisms of host iron piracy to scavenge this metal during infection [3].

Iron exists in the reduced ferrous (Fe^2+^) or oxidized ferric (Fe^3+^) forms. The Fe^2+^/Fe^3+^ redox potential makes it extremely versatile when incorporated into proteins as a catalytic center or electron carrier, and it is essential in numerous biological processes. Although abundant in nature, iron does not normally occur in its biologically active ferrous form. Under aerobic conditions, Fe^2+^ is unstable and generates Fe^3+^ and reactive oxygen species (ROS) via the Fenton reaction, the latter of which can damage lipids, DNA, and proteins [4]. Conversely, ferric iron is poorly water-soluble and requires specialized proteins to facilitate its mobilization and maintain intracellular reservoir (lactoferrin and transferrin for transport, ferritin for storage). However, the most abundant form of iron in vertebrates is bound within a porphyrin ring as ferriprotoporphyrin IX (heme) [5], most of which is bound to intracellular proteins (hemoglobin, myoglobin, cytochromes). Hemoglobin from lysed erythrocytes is bound by haptoglobin, whereas free heme is bound to hemopexin and, to a lesser extent, to albumin, under which circumstances it may become available to extracellular pathogens [6]. Many pathogens thus exploit host heme as a nutrient iron source [5,7,8].

In the lungs, iron is stored in ferritin, macrophages sequester heme from senescent erythrocytes, take and store iron from transferrin, and clear inhaled iron-containing particulate matter. Likewise, neutrophils release iron regulatory molecules helping sequester free iron [9–11]. Human lung iron levels range between 0.4–0.9 mg/g, and ~10 μg iron are exposed to lungs daily at a normal rate of breathing from the atmosphere [12]. Exposure to higher iron levels through inhalation of iron-containing particulate matter provides catalytically active iron resulting in tissue damage. Moreover, alveolar hemorrhaging observed in several lung disease conditions leads to increased iron levels. As a consequence, strong evidence for dysregulated iron homeostasis exists in major respiratory diseases including chronic obstructive pulmonary disease (COPD) [1,2,13–15]. COPD is a complex progressive condition characterized by chronic airway inflammation, associated with airway remodeling, alveolar destruction and persistent airflow obstruction [16]. Cigarette smoking, the single greatest risk factor for developing COPD [17], contains iron particulates inducing intracellular iron accumulation [13,14]. Elevated levels of iron, ferritin, and oxidative stress are found in the lungs of smokers, where alveolar macrophages increase iron loading [13,14,18–20]. Similarly, the COPD airways present altered levels of iron-binding proteins, increased iron loading by alveolar macrophages, and high rates of polymorphisms in genes associated with iron regulation [21–28]. Given that iron overload increases the virulence of numerous pathogens [29,30], dysregulation of iron homeostasis in the COPD lungs may also facilitate persistence by opportunistic pathogens in the lower airways [2,6,11].

**Figure 1.**
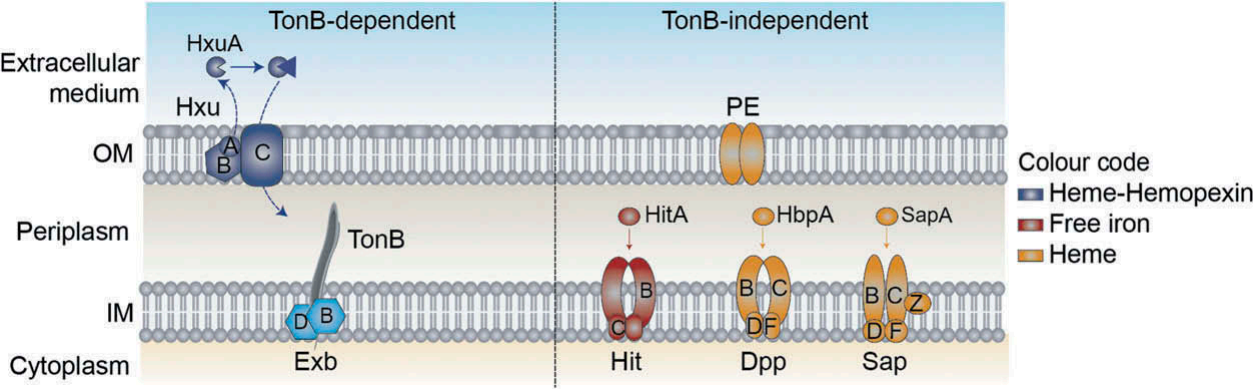
**Schematic representation of NTHi heme uptake systems considered in this study**. HxuCBA is a TonB-dependent system involved in heme-hemopexin binding. HxuC is a receptor and HxuAB is a two-partner secretion system. HxuA is exposed at the cell surface, and leads to heme release and its capture by HxuC. SapABCDFZ, HbpA-DppBCDF and PE are TonB-independent systems. SapA and HbpA are periplasmic proteins that bind heme and are linked to the inner membrane ABC transporters SapBCDFZ and DppBCDF, respectively. PE is an outer membrane protein that binds heme as a dimer. The HitABC free iron uptake system is also shown. HitA is a periplasmic protein that binds free iron linked to the HitBC transporter located at the inner membrane. Heme-iron sources are color-coded.

Nontypeable *Haemophilus influenzae* (NTHi) is typically a commensal of the human upper airways, but also a common opportunistic pathogen in the lower airways of patients with COPD [31–35]. NTHi persistence within the COPD lungs contributes to airway inflammation that results in worsening of symptoms and promotes disease progression [33,36–38]. NTHi is incapable of synthesizing heme, but possesses a ferrochelatase that reversibly inserts iron into protoporphyrin IX (PPIX) to form heme [39,40]. Thus, it requires either iron in the presence of PPIX or exogenous heme to grow aerobically [41]. NTHi presents a variety of heme uptake-binding systems including TonB-dependent outer membrane systems that bind heme or hemoproteins (Hup [42], HemR [43], Hgps [44–50], HxuCBA [51–56]), and TonB-independent periplasmic proteins that bind heme and are linked to inner membrane ABC transporters (SapABCDFZ, HbpA-DppBCDF [57–62]). NTHi also stores and shares heme by using the outer membrane protein E (PE) [63] (Figure 1). Information on the contribution of heme uptake to NTHi respiratory infection is scattered [47,50,55,60,64], and a global understanding is lacking.

Existing evidence prompted us to hypothesize that pathological disruption of lung iron homeostasis and bacterial heme auxotrophy may have evolved NTHi toward the coordinated action of its repertoire of heme scavenging mechanisms, to evade nutritional immunity and facilitate the host-pathogen interplay. In this study, HxuCBA, PE, SapABCDFZ, and HbpA-DppBCDF were selected as representative *H. influenzae* heme binding systems at the outer membrane and within the periplasm (Figure 1). Systematic bacterial gene inactivation and phenotypic assessment revealed their relative contribution to NTHi airway infection, and their ability to bind heme was investigated *in silico*.

## Materials and methods

### Computational modeling

Docking of heme B to HbpA, SapA, PE, and HxuA was performed using the HADDOCK2.2 (High Ambiguity-Driven protein-protein DOCKing) server [65,66]. For HbpA and SapA, the I-TASSER server [67,68] was used to generate homology models based on the crystal structure of HbpA from *Haemophilus parasuis* in its ligand-bound form (PDB code 3M8U) [62]. For both proteins, the best scoring I-TASSER model was used for the docking experiment. To drive the docking, residues with 60% ligand binding probability as estimated by the COACH function within the I-TASSER suite [69,70] were included as part of an initial estimation of the location of the binding site (residues 61–64, 155, 280, 396, 398, 445–447, and 537 for HbpA; residues 54–55, 458–460, and 462 for SapA). In addition to full-length SapA, heme docking was also performed after removing residues 140–158. For PE, heme was docked to the crystal structure of the PE-dimer (3ZH5). Residues from both monomers within the predicted heme binding pocket (59, 65, 67, 94, 96, 98, 101, 103, and 107) were used to drive the docking. For HxuA, heme was docked to the HxuA crystal structure (PDB code 4RM6), and residues in a manually identified putative binding pocket (450, 452, 464, 516, 526, 556, 560, and 572) were used to drive the docking. Docking solutions were evaluated based on the HADDOCK score and Z-score (the number of standard deviations the HADDOCK score of a given solution is separated from the mean of all clusters). For HbpA, truncated SapA, and PE, a single statistically significant solution cluster was obtained (p < 0.05) whereas for HxuA, two equally good solutions were obtained. For full-length SapA, a second solution (cluster 4) with a non-significantly different HADDOCK score from the top solution was obtained. However, this was discarded due to the significantly higher Z-score (−0.2 compared to −1.7 for the top solution). Detailed docking statistics are given in Table S4. The top solution in each cluster was further analyzed in PyMOL (PyMOL Molecular Graphics System, version 2.0, Schrödinger, LLC), and LigPlot^+^ [71], which were also used to generate figures.

### Bacterial strains, plasmids, and growth media

Strains and plasmids used in this study are described in Table 1 and Table S1. NTHi strains were grown for 16 h, at 37°C, 5% CO_2_, on chocolate agar (Biomérieux), Mueller Hinton-Fastidious agar (MH-F) (Biomérieux), *Haemophilus* Test Medium agar (HTM, Oxoid), or Brain-Heart Infusion agar (BHI, Oxoid). BHI was supplemented with 10 μg/ml nicotinamide adenine dinucleotide (NAD, factor V) (Sigma-Aldrich) (referred to as BHI-NAD or heme-deficient medium). Alternatively, HTM and BHI were supplemented with both NAD and 10 μg/ml heme (factor X) (Sigma-Aldrich) (referred to as sHTM and sBHI). Liquid cultures were grown in heme-deficient medium, sBHI or, alternatively, in a chemically defined minimal medium (CDMM) supplemented with 50 μM cystine (Sigma-Aldrich) or 50 μM glutathione disulfide (GSSG) (Sigma-Aldrich) (Table S2 [62,72,73]). GSSG (20 mM) was prepared in sterile water; 20 mM cystine was prepared in 1M HCl, filtered and maintained at 4°C until use. Erythromycin (Erm) at 11 μg/ml or spectinomycin (Spec) at 50 μg/ml was used when required. *Escherichia coli* was grown on Luria Bertani (LB) agar at 37°C, supplemented with ampicillin at 100 μg/ml, Erm at 150 μg/ml, or Spec at 50 μg/ml, when indicated.

**Table 1.** Bacterial strains used in this study.

Strain	Description	Source
***H. influenzae***		
NTHi375	Wild-type, otitis media clinical isolate	[117]
NTHi375Δ*hxuCBA*-P682	*hxuCBA::spec*, Spec^R^	This study
NTHi375Δ*sapA*-1665	*sapA::ermC::ermC*, Erm^R^	This study
NTHi375Δ*hbpA*-P896	*hbpA::spec*, Spec^R^	This study
NTHi375Δ*hpe*-P897	*hpe::spec*, Spec^R^	This study
RdKW20	Laboratory strain, capsule-deficient serotype d	[97]
RdKW20Δ*hxuCBA*-P683	*hxuCBA::spec*, Spec^R^	This study
RdKW20Δ*sapA*-P578	*sapA::ermC::ermC*, Erm^R^	This study
RdKW20Δ*hbpA*-P885	*hbpA::spec*, Spec^R^	This study
RdKW20Δ*hitBC*-P804	*hitBC::ermC*, Erm^R^	This study
NTHi3655*luxABCDE*-P845	Luminiscent strain derived from isolate NTHi3655. It contains a copy of the *luxABCDE* operon inserted in the genome.	[63]
***E. coli***		
TOP10	Used for cloning assays	Thermofisher Scientific
SW102	Derived from DY380, it contains a defective λ prophage with the recombination proteins *exo*, *bet*, and *gam* being controlled by the temperature-sensitive repressor *cI*857	[75]

### Generation of *H. influenzae* mutant strains

Plasmids, primers, and construct design are shown in Tables S1, S3, and Fig. S1A. Briefly, a DNA fragment containing each gene/operon and its respective flanking regions was PCR amplified with *Phusion* polymerase (ThermoScientific) using NTHi375 genomic DNA as template and primers gene+flanking region-F1 and gene+flanking region-R1, and cloned into pJET1.2/blunt (ThermoScientific) or pGEMT-easy (Promega), generating plasmids pJET1.2-*hxuCBA*, pGEMT-*sapAB*, pJET1.2-*hbpA,* and pJET1.2-*hpe*. To generate pJET1.2-*hitABC*, two DNA fragments of the *hitABC* operon were independently amplified using primers *hitABC*-F1/*hitABC*-R2 and *hitABC*-F2/*hitABC-*R1, respectively, *Kpn*I digested and ligated, rendering a product with a 853 bp deletion of the *hitB* gene 3´ end and a 200 bp deletion of the *hitC* gene 5´ end, which was further amplified with primers *hitABC*-F1 and *hitABC*-R1 and cloned into pJET1.2 to generate pJET1.2-*hitABC*. An Erm-based disruption strategy was used to generate Δ*sapA* and Δ*hitBC* strains. pGEMT-*sapAB* was disrupted by inverse PCR with *Phusion* polymerase, using primers *sapAB*-F2 and *sapAB*-R2. A *sapA* gene internal fragment of 168 bp was replaced by a blunt-ended ErmC resistance cassette excised by *Sma*I digestion from pBSLerm [74], generating pGEMT-*sapA::ermC*, which was used as a template to amplify the *sapA::ermC* disruption cassette with primers *sapAB*-F1 and *sapAB*-R1. pJET1.2-*hitABC* was *Kpn*I digested and used as cloning vector for a *Kpn*I blunt-ended ErmC resistance gene excised by *Kpn*I digestion from pBSLerm, generating pJET1.2-*hitBC::ermC*, which was used as a template to amplify the *hitBC::ermC* disruption cassette with primers *hitABC*-F1 and *hitABC*-R1. Alternatively, a Spec-based disruption strategy was used to generate Δ*hxuCBA*, Δ*hbpA,* and Δ*hpe* strains. To do so, a Spec resistance gene was independently PCR amplified from pRSM2832 using gene-specific mutagenic primer pairs *hxuCBA*-F2/*hxuCBA*-R2, *hbpA*-F2/*hbpA*-R2, and *hpe*-F2/*hpe*-R2 [75]. *E. coli* SW102 cells were prepared for recombineering, separately co-electroporated with pJET1.2-*hxuCBA*, pJET1.2-*hbpA* or pJET1.2-*hpe* (Amp^r^) (50 ng), and with each gene-specific mutagenic cassette (Spec^r^) (200 ng) [76]. Mutagenized clones containing pJET1.2-*hxuCBA::spec*, pJET1.2-*hbpA::spec* and pJET1.2-*hpe::spec* were selected on LB agar with Amp_100_ and Spec_50_. These plasmids were used as templates to amplify the *hxuCBA*::spec, *hbpA*::spec and *hpe*::spec disruption cassettes with primer pairs *hxuCBA*-F1/*hxuCBA*-R1, *hbpA*-F1/*hbpA*-R1, and *hpe*-F1/*hpe*-R1, respectively. Disruption cassettes were independently used to transform NTHi375 or Hi RdKW20 by using the MIV method [77]. Transformants were selected on sBHI agar with Erm at 11 μg/ml to obtain Δ*sapA* and Δ*hitBC* strains, and on sBHI agar with Spec at 50 μg/ml to obtain Δ*hxuCBA*, Δ*hbpA,* and Δ*hpe* strains. All mutants were confirmed by PCR and sequencing. The *sapABCDFZ* and *hbpA*-HI0854/NTHI1022 are gene clusters containing 6 and 2 genes, respectively. Expression of the *sapBCDFZ* and HI0854/NTHI1022 genes in NTHi375Δ*sapA* and NTHi375Δ*hbpA* strains was tested by RT-PCR (see below), being comparable to that found in the wild type (WT) strain (Fig. S1B).

### Bacterial growth

Strains grown on chocolate agar were used to generate bacterial suspensions normalized in PBS to OD_600_ = 1. Suspensions were spread on 20 ml BHI agar in the presence of sterile X + V factor disks (Oxoid), and grown during 20 h at 37°C. Growth diameter was measured on at least three independent occasions (*n* ≥ 3). Alternatively, normalized bacterial suspensions were diluted to OD_600_ = 0.05 in 2 ml sBHI, and 200 µl aliquots were transferred to individual wells in 96-well microtiter plates (Falcon). Plates were incubated with agitation at 37°C for 6 h in a Multiskan device (ThermoScientific), and OD_636_ was monitored every 15 min. Each growth curve was corrected to its blank values (sBHI). Independently, strains were grown on chocolate agar; biomass was inoculated in 7 ml sBHI and incubated for 8 h at 37°C with shaking (200 r.p.m.). Cultures were centrifuged (10 min, 4000 r.p.m.) and suspensions were normalized to OD_600_ = 1 in PBS. Then, 180 μl of CDMM supplemented with either 50 μM cystine or 50 μM GSSG were transferred to individual wells in 96-well microtiter plates (Sarstedt). Thereafter, 20 μl of the previously prepared bacterial suspensions were added to each well. Plates were incubated in a SpectraMAX 340 microplate reader at 37°C, and OD_600_ was recorded every 30 min for 8 h. In all cases, bacterial growth was monitored in triplicate at least three times (*n* ≥ 9).

### Heme binding to the bacterial surface

As previously stated [63], strains were grown on chocolate agar, inoculated in 5 ml heme-deficient medium and grown for 8 h, at 37°C with shaking (200 r.p.m.). Next, 100 μl culture aliquots were inoculated into 20 ml sBHI and grown for 16 h under the same conditions. Normalized bacterial suspensions were collected by centrifugation (10 min, 4000 r.p.m.), washed three times with PBS, and resuspended in 5 ml PBS. For semi-quantitative determination of bacterial heme, 5 ml PBS suspensions were 2-fold serially diluted in PBS, 100 µl aliquots spotted on PVDF membranes, and heme was detected by an enhanced chemiluminescence (ECL) Western Blot detection kit (Pierce).

### Co-culture of NTHi3655luxABCDE with heme donors

NTHi3655*luxABCDE* (recipient) was co-cultured with *H. influenzae* heme donors [63]. Donor strains were grown on chocolate agar, inoculated in 5 ml heme-deficient medium, and grown for 8 h with shaking (200 r.p.m.). Thereafter, 100 µl aliquots of these cultures were added to 20 ml sBHI and grown for 16 h under the same conditions. To generate heme starvation, NTHi3655*luxABCDE* was grown on chocolate agar, inoculated in 20 ml heme-deficient medium, and grown overnight with shaking (200 r.p.m.). All cultures were then recovered by centrifugation (10 min, 4000 r.p.m.), washed twice in BHI, and resuspended in 10 ml BHI. For co-cultures, appropriate volumes of each suspension (donor and recipient) containing identical number of colony forming units (CFU) were mixed in 5 ml heme-deficient medium generating a starting co-culture with OD_600_ = 0.1, to be incubated for 6 h. For recipient controls, similar NTHi3655*luxABCDE* cultures were prepared and grown in sBHI or heme-deficient medium (positive and negative controls, respectively). Luminescence was monitored every hour for 6 h in a 1,450 MicroBeta TriLux counter (PerkinElmer), using 100 µl bacterial culture aliquots and 96-well black plates (Uniplate®, Whatman). Experiments were performed in triplicates at ≥ 3 independent occassions (*n* ≥ 9).

### Bacterial susceptibility to antimicrobials

Antibiotic minimal inhibitory concentrations (MICs) were determined by microdilution using commercial panels (STRHAE2; Sensititre), and interpreted by following the criteria of the Clinical Laboratory Standards Institute (CLSI) [78]. For polymyxin E (PxE), Etests (Biomérieux) on MH-F agar were performed: bacteria were grown on chocolate agar for 8 h, inoculated in 7 ml of heme-deficient medium or sBHI, and grown for 12 h at 37°C, 200 r.p.m. Cultures were centrifuged (10 min, 4000 r.p.m.), suspensions normalized to ~0.5 McFarland (equivalent to OD_600_ = 0.063) in saline solution, and used as inoculum on MH-F agar (*n* = 2). To monitor susceptibility to H_2_O_2_, bacteria grown on chocolate agar for 8 h were inoculated in 7 ml of sBHI, and grown for 12 h at 37°C, 200 r.p.m. Cultures were centrifuged (10 min, 4000 r.p.m.), suspensions were normalized to OD_600_ = 1 (~7×10^8^ CFU/ml) in PBS and plated on sHTM agar on large Petri dishes (20 cm diameter; 80 ml sHTM agar/plate; 1.8 ml suspension/plate). H_2_O_2_ at 9.8 M (Sigma-Aldrich) was used as stock solution to be diluted in sterile distilled water. Sterile paper disks soaked on 10 μl H_2_O_2_ containing 25 or 50 μmol were located on the sHTM agar and incubated for 20 h at 37°C. Results are shown as diameter of bacterial growth inhibition (mm) around H_2_O_2_ disks (means ± SD). Assays were performed at ≥ 3 independent occassions (*n* ≥ 3).

### RNA extraction and real-time quantitative PCR (RT-qPCR)

Two to 5 colonies of strains grown on chocolate agar were inoculated in 20 ml sBHI, grown for 11 h, diluted into 40 ml fresh sBHI to OD_600_ = 0.05, and grown to OD_600_ = 0.5–0.6 (named as conventional sBHI cultures). Alternatively, bacterial biomass was collected from chocolate agar, inoculated in 5 ml heme-deficient medium and grown for 8 h. Next, 100 μl aliquots were inoculated into 20 ml sBHI and grown overnight (37°C, 5% CO_2_, 200 r.p.m.). Overnight cultures were collected by centrifugation (10 min, 4000 r.p.m.), washed 3 times in PBS, resuspended in 5 ml PBS, diluted to OD_600_ = 0.05 in 40 ml fresh BHI-NAD or sBHI, and grown to OD_600_ = 0.4–0.5 [63]. Bacterial total RNA was isolated using TRIzol reagent (Invitrogen), and total RNA quality evaluated using RNA 6,000 Nano LabChips (Agilent 2,100 Bioanalyzer, Santa Clara, CA). All samples had intact 23S and 16S ribosomal RNA bands. Reverse transcription (RT) was performed using 1 µg of RNA by PrimerScript RT Reagent kit (Takara). PCR amplification was performed by using SYBR Premix Ex Taq II (Tli RNaseH Plus) (Takara). Fluorescence was analyzed with AriaMx Real-Time PCR System (Agilent Technologies). The comparative threshold cycle (Ct) method was used to obtain relative quantities of mRNAs that were normalized using 16S ribosomal RNA (*16SrRNA*) as an endogenous control. Primers were designed with Primer3 software (Table S3). All cultures were grown at least three times, and samples were processed with technical triplicates (*n* ≥ 3).

### Cell culture and bacterial infection

A549 type II human pneumocytes (ATCC CCL-185) were maintained and seeded as described [79–86]. For infection, PBS-normalized bacterial suspensions (OD_600_ = 1) were prepared by using NTHi strains grown on chocolate agar. Alternatively, bacteria grown on chocolate agar were inoculated in 7 ml heme-deficient medium or sBHI, and grown for 12 h at 37°C, 200 r.p.m. Cultures were centrifuged (10 min, 4000 r.p.m.), suspensions normalized to OD_600_ = 1 in PBS, and then used as infecting inoculum. A multiplicity of infection (MOI) of ~100:1 was used. To monitor adhesion, cells were infected for 30 min. This assay does not completely exclude a possible internalization of some bacteria, and experimental conditions were previously set to mainly monitor adhesion [84]. For invasion testing, cells were incubated with bacteria for 2 h, washed 3 times with PBS, incubated for 1 h with RPMI 1640 medium containing 10% FCS, 10 mM Hepes, and 200 µg/ml gentamicin. After infection, cells were lysed with PBS-saponin 0.025%, 10-fold diluted and plated on sBHI agar. All infections were performed in triplicates at ≥ 3 independent occassions (*n* ≥ 9). Results are expressed as CFU/well.

### Secretion of IL-8

Bacteria grown on chocolate agar were collected with PBS, suspensions normalized to OD_600_ = 1 and used for 2 h infection with a MOI of ~100:1. Cells were washed three times with PBS, and incubated for 6 h in RPMI 1640 medium containing 10% FCS, 10 mM Hepes, and 100 µg/ml gentamicin. Supernatants were collected from the wells, cell debris was removed by centrifugation and samples were frozen at −80°C. IL-8 levels in supernatants were measured by ELISA (Abnova KA0115) with sensitivity <2 pg/ml. Infections were performed in duplicate and at least twice (*n* = 4). Results on IL-8 are expressed in pg/ml.

### NTHi mouse lung infection

A mouse model of NTHi lung infection was used as described previously [86]. CD1 female mice were purchased from Charles River Laboratories (France), housed under pathogen-free conditions at the Institute of Agrobiotechnology facilities (registration number 365 ES/31-2016-000002-CR-SU-US), and used at 22–25 g (6–7 weeks). Animal handling and procedures were in accordance with the current European (Directive 2010/63/EU) and National (Real Decreto 53/2013) legislations, following the FELASA and ARRIVE guidelines, with the approval of Universidad Pública de Navarra (UPNa) Animal Experimentation Committee, and in accordance with the Helsinki declaration. Bacteria grown on chocolate agar were recovered in PBS generating normalized suspensions (~5 × 10^9^ CFU/ml). Next, 20 µl suspensions (~1 × 10^8^ CFU/mice) were placed at the entrance of the nostrils until complete inhalation, in mice previously anesthetized with ketamine-xylazine (3:1). At 24 or 48 h post-infection (hpi), mice were euthanized using cervical dislocation. Bronchoalveolar lavage fluid (BALF) samples were obtained by perfusion and collection of 0.7 ml of PBS, with help of a sterile 20G (1.1 mm diameter) VialonTM intravenous catheter (Becton-Dickinson) inserted into the trachea. Recovered BALF was serially 10-fold diluted in PBS, and plated in triplicate on sBHI agar to determine the number of viable bacteria. By following standardized published procedures [87], we considered that we could have a minimum of 3.3 CFU in 1 ml of sample without detecting bacteria (limit of detection <3–4 CFU/ml BALF), rendering log_10_ = 0.52. The left lung was individually weighed in sterile bags (Stomacher80, Seward Medical) and homogenized 1:10 (wt/vol) in PBS. Each homogenate was serially 10-fold diluted in PBS and plated in triplicate on sBHI agar to determine the number of CFU per lung. Infections were performed in groups of at least five mice per strain and time point (*n* ≥ 5).

### Statistical analysis

In all cases, p < 0.05 value was considered statistically significant. Analyses were performed using Prism software, version 7 for Mac (GraphPad Software) statistical package, and are detailed in each figure legend.

## Results

### Structural basis of heme binding to the HbpA, SapA, PE, and HxuA proteins

First, we investigated the ability of HxuA, HbpA, PE, and SapA to bind heme *in silico* using the HADDOCK (High Ambiguity-Driven Protein DOCKing) server [65,66]. HxuA is part of the heme-hemopexin heme transport system which consists of the HxuC and the two-partner secretion system HxuBA. HxuA is exposed at the cell surface, and its interaction with hemopexin leads to heme release and its capture by HxuC [51–56]. PE is an outer membrane lipoprotein, the dimer of which has been proposed to bind heme [63]; and SapA and HbpA are Cluster C substrate-binding proteins (SBP) that locate at the periplasm and act in concert with the SapBCDFZ and DppBCDF inner membrane ABC transporters, respectively [57,59–62]. Docking results for all four proteins are summarized in Table S4.

Since there are no crystal structures of HbpA and SapA, homology models were constructed based on the crystal structure of HbpA from *H. parasuis* (*Hp*HbpA) in its glutathione-bound form (PDB code 3M8U) [62] using I-TASSER [67,68]. The sequence identity for the structurally aligned region was 74% and 30% for HbpA and SapA, respectively. Despite the lower sequence identity for SapA, a sequence alignment with HbpA, *Hp*HbpA and the structurally homologous dipeptide-binding protein DppA from *E. coli* (*Ec*DppA) [88] confirmed high sequence similarity (Fig. S2), supporting *Hp*HbpA as a good structural template for SapA as well as HbpA. Ligand binding site residues predicted by COACH [69,70], a ligand-binding prediction server integrated into I-TASSER suite, was used to drive the docking.

**Figure 2.**
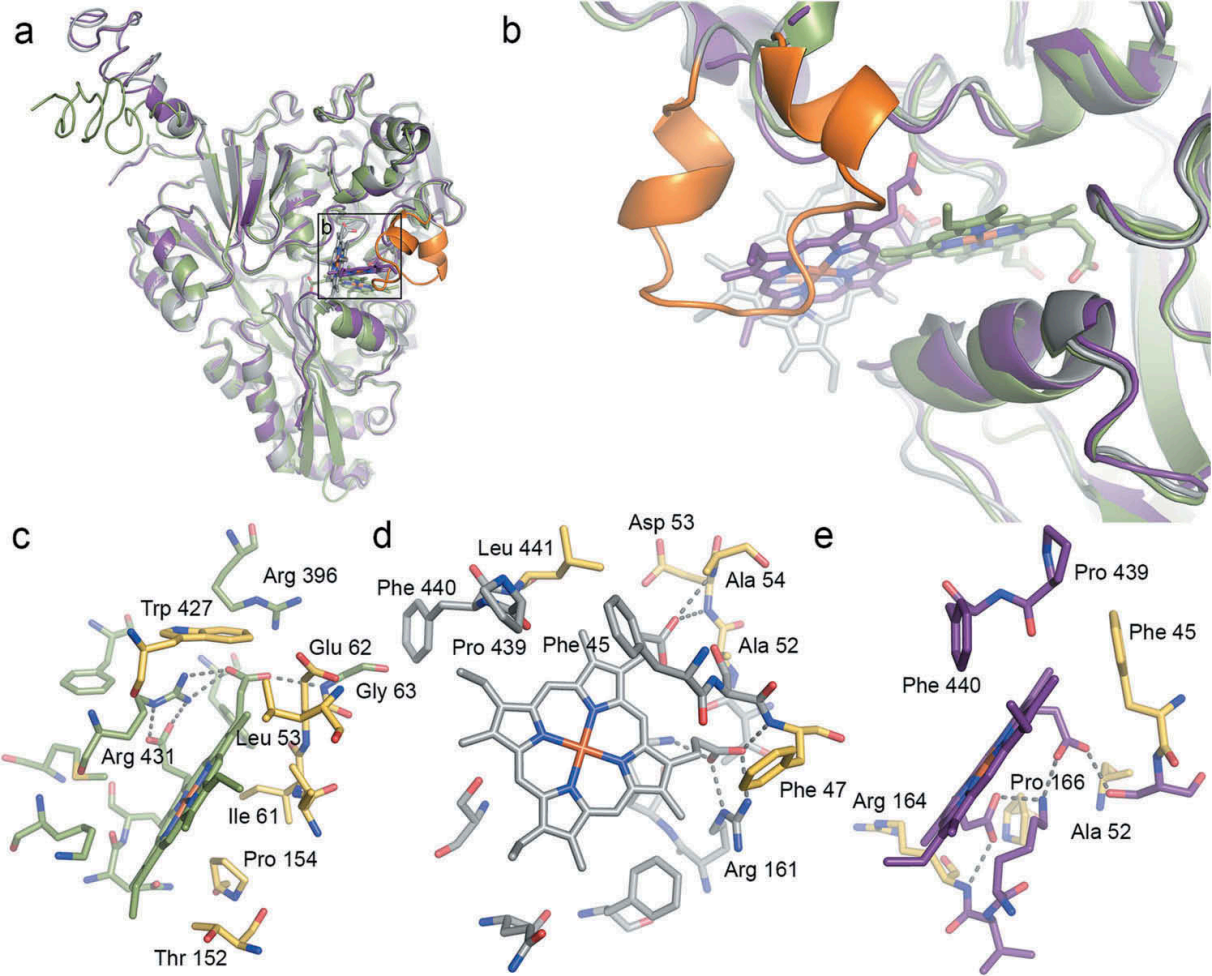
**Structural prediction of heme binding to HbpA and SapA**. HADDOCK was used to dock heme B to homology models of HbpA (green), full-length SapA (grey) and truncated SapA (purple) in which a non-conserved loop consisting of residues 140–158 has been removed. The truncated residues are colored orange in full-length SapA. **(a)** Overlay of the best scoring docking solutions for HbpA, SapA and truncated SapA. **(b)** Zoom-in on predicted heme binding sites showing how HbpA binds heme deeper into the ligand-binding pocket. In full-length SapA, the non-conserved loop (orange) prevents heme binding into the same pocket whereas in truncated SapA, the pocket is more accessible. **(c-e)** LigPlot^+^ analysis of the predicted heme binding site in HbpA **(c)**, full-length SapA **(d)** and truncated SapA **(e)** with residues lining the binding sites in stick representation. Predicted hydrogen bonds are showed as dashed lines. Residues that are common for all three binding sites are colored yellow.

For HbpA, the highest scoring docking solution suggests heme binding at a cleft between the N-terminal and C-terminal domains (Figure 2(a–e)), similar to what is seen for binding of glutathione and dipeptide to *Hp*HbpA and *Ec*DppA respectively (Fig. S3A-B). In SapA, heme docks in a similar location but at a different angle due to the presence of a longer loop (residues 140–158, in orange in Figure 2(a,b)). This loop extension is not conserved in HbpA, *Hp*HbpA and *Ec*DppA, and thus seems to be a unique feature of SapA (Fig. S2). Since the position of this loop must be regarded as speculative due to its absence in the homology modeling template, we removed it from the SapA homology model and repeated the docking experiment. In the absence of the extended loop, the predicted heme binding mode is closer as to what is seen for HbpA, although the site is closer to the protein surface.

Analysis of the heme binding sites using LigPlot^+^ [71] shows predicted hydrogen bonds between heme propionates and positively charged side chains or main chain nitrogens in all three cases, as well as the presence of hydrophobic residues lining the pockets and providing van der Waals interaction with the rest of the heme group (Figure 2(c-e) and Fig. S3C-D). Despite the differences in heme binding mode, several residues are common for all three binding sites (colored yellow in Figure 2(c–e)).

For HbpA, the suggested site is supported by what has been previously predicted [89] and corresponds well to a very recent study where heme was docked to *Hp*HbpA, OppA from *H. influenzae* (*Hi*OppA) and NikA from *E. coli* (*Ec*NikA) [90]. In the latter study, heme was suggested to bind in a site that was distinct but close to the site for other ligands (glutathione for *Hp*HbpA, peptide for *Hi*OppA, and butane-1,2,4-tricarboxylate-chelated nickel for *Ec*NikA) and the ability of bind heme and other ligands simultaneously was demonstrated using surface plasmon resonance. Our docking results reveal a similar scenario, with heme binding close to- but not overlapping with the binding site for glutathione in *Hp*HbpA (Fig. S3).

From the crystal structure of PE, it was suggested that heme could bind in a well-defined pocket formed at the PE dimer interface. Manual docking of heme into this pocket showed that it should be able to accommodate heme but no additional evaluation was performed [63,91]. Here we further explored heme binding to PE by docking heme into the predicted heme-binding pocket. Our results show that heme fits very well with predicted hydrogen bonds formed between heme propionates and polar residues within one of the PE monomers (Asn98, Asn101, and Thr105), as well as van der Waals interactions with hydrophobic residues on both sides of the pocket (Ile65 and Ile96) (Fig. S4A-C).

While HxuC reconstitution in *E. coli* previously showed its ability to act independently as a functional free heme receptor [53], direct heme binding to HxuA has not been demonstrated. Nevertheless, upon examination of the HxuA crystal structure (PDB code 4RM6) [92], we identified a pocket at the HxuA surface, close to the hemopexin binding site, that showed similarities with the predicted heme binding site in HbpA as indicated above, most notably the presence of several positively charged residues that may interact with heme propionates. The ability of this pocket to accommodate a heme molecule was investigated using HADDOCK, resulting in two solutions with similar docking scores (Table S4). As shown in Fig. S4D-E, heme fits well into this pocket, with the two solutions differing by the heme propionates pointing into or out of the pocket. In both solutions, several positively charged residues (Arg and/or Lys) are well positioned to interact with heme propionates via hydrogen bonds.

In summary, our computational studies provide plausible 3D models for how HbpA, SapA, PE, and HxuA interacts with heme.

### Expression of NTHi heme binding protein encoding genes and their response to heme availability

*H. influenzae* genes encoding heme-binding proteins are transcribed during acute infection [93]. Multiple heme acquisition systems may counteract NTHi heme auxotrophy and human iron nutritional immunity, but could also lead to redundancy. Based on these assumptions, we asked if the systems under analysis could be inter-related to preserve bacterial fitness or prevent heme overloading toxicity, and if such relationship may occur at the gene expression level.

The NTHi375 strain was employed to independently generate *hxuCBA, sapA, hbpA,* and *hpe* mutants (gene accession numbers NF38_04090/NF38_04085/NF38_04080, NF38_08010/NF38_08015/NF38_08020/NF38_08025/NF38_08030/NF38_08035, NF38_00680, and NF38_04540) (Table 1). Initial characterization of the generated mutants rendered the following features: (i) growth in sBHI, with slightly longer lag phase for the *hxuCBA, hbpA,* and *hpe* mutants than for the WT strain (Fig. S5A). (ii) Aerobic growth on BHI agar around X + V factor disks comparable to that of the WT strain, except for Δ*hxuCBA,* whose growth showed a trend to be lower (NTHi375 WT, 138 ± 17 mm; NTHi375Δ*hxuCBA*, 122 ± 18 mm) (Fig. S5B). (iii) Anaerobic growth on BHI agar around V factor disks, and antibiotic susceptibility patterns (Table S5) similar to the WT strain. (iv) Inactivation of heme acquisition systems could modify bacterial iron levels and subsequently, iron reactivity with H_2_O_2_ via the Fenton reaction. To prove this phenomenon, inactivation of the *sapA* and *hbpA* genes increased growth inhibition around paper disks soaked with H_2_O_2_ 25 and 50 μmol compared to the WT strain (Fig. S5C).

**Figure 3.**
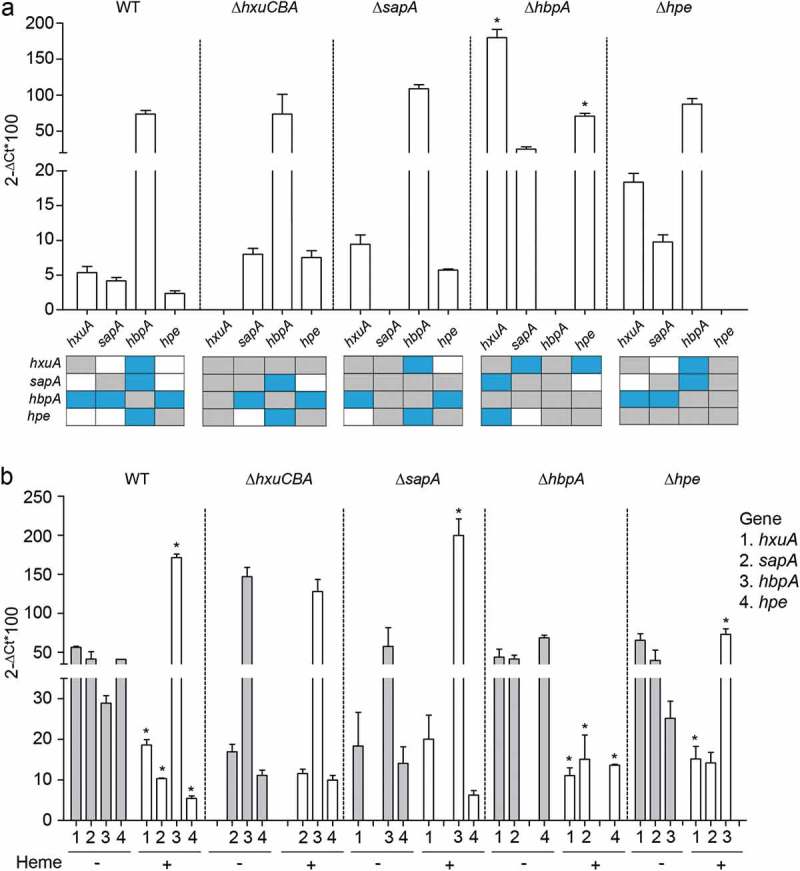
**Expression of NTHi heme acquisition systems. (a)** Expression of the *hxuA, sapA, hbpA* and *hpe* genes in NTHi375 WT, Δ*hxuCBA*, Δ*sapA*, Δ*hbpA,* and Δ*hpe* strains grown in sBHI. Expression of the *hbpA* gene was higher than that of *hxuA, sapA,* and *hpe* (WT strain, higher expression of *hbpA* than that of *hxuA* (p < 0.05), *sapA* and *hpe* (p < 0.01); NTHi375Δ*hxuCBA*, higher expression of *hbpA* than that of *sapA* and *hpe* (p < 0.0005); NTHi375Δ*sapA*, higher expression of *hbpA* than that of *hxuA* and *hpe* (p < 0.0005); NTHi375Δ*hpe*, higher expression of *hbpA* than that of *hxuA* (p < 0.05) and *sapA* (p < 0.0005)). Expression of the *hxuA* and *hpe* genes was higher in NTHi375Δ*hbpA* than in the WT strain (p < 0.0001 and p < 0.01, respectively). Bottom panel: schematic representation of each gene expression compared to each other, in the WT and mutant strains. Color code: blue, statistically significant differences; gray, not determined; white, no significant differences. Data are shown as mean ± SEM. Statistical comparisons of the means were performed with two-way ANOVA (Tukey´s multiple comparisons test). **(b)** Gene expression and heme sensing. In NTHi375 WT, Δ*hbpA* and Δ*hpe* strains, *hxuA, sapA,* and *hpe* gene expression was higher in heme-restricted medium than in sBHI (WT: *hxuA* (p < 0.001), *sapA* (p < 0.01), *hpe* (p < 0.001); NTHi375Δ*hbpA: hxuA* (p < 0.01), *sapA* (p < 0.05), *hpe* (p < 0.0001); NTHi375Δ*hpe, hxuA*, p < 0.05). In NTHi375 WT, Δ*sapA* and Δ*hpe* strains, expression of *hbpA* was lower upon heme depletion (WT, p < 0.0001; NTHi375Δ*sapA*, (p < 0.001); NTHi375Δ*hpe*, p < 0.05). Data are shown as mean ± SEM. Statistical comparisons of the means were performed with two-way ANOVA (Sidak´s multiple comparisons test).

NTHi375 WT and mutant strains were used to determine the expression of the *hxuA, sapA, hbpA,* and *hpe* genes. First, conventional sBHI cultures were employed. The *hbpA* gene was highly expressed in all cases, with higher expression than that of the *hxuA, sapA,* and *hpe* genes. Inactivation of the *hxuA, sapA,* and *hpe* genes did not alter *hbpA* gene expression. However, expression of the *hxuA, sapA,* and *hpe* genes showed a consistent trend to be higher in the mutants than in the WT strain, being significant for the *hxuA* and *hpe* genes when comparing their expression in the WT and NTHi375Δ*hbpA* strains (Figure 3(a)).

Bacteria sense heme as means to alert pathogens upon contact with vertebrate tissues, thus modulating expression of systems involved in heme acquisition and metabolism [29,94]. Expression of genes encoding most heme uptake systems increases when iron or heme are restricted [95,96]. We next asked if gene expression responsive to heme availability occurs in a coordinated manner. NTHi375 WT and mutant strains were grown in heme-deficient medium or in sBHI for RNA purification and qRT-PCR analyses. In NTHi375 WT, Δ*hbpA,* and Δ*hpe* strains, expression of the *hxuA, sapA,* and *hpe* (the latter in WT and Δ*hbpA* strains) genes was higher in heme-restricted medium than in sBHI. In NTHi375 WT, Δ*sapA,* and Δ*hpe* strains, expression of the *hbpA* gene was lower upon heme depletion. Lastly, *hxuCBA* gene inactivation seemed to hamper the effect of heme availability on gene expression (Figure 3(b)).

Overall, these results support the notion that NTHi heme acquisition machinery may act in a coordinated manner, where HbpA seems to play a prominent role, likely followed by PE, and HxuCBA and SapABCDFZ participate in heme sensing, in turn having an impact on the expression of other uptake systems.

## SapA, HbpA, and HxuA participate in heme donation to heme-starved NTHi bacterial cells

**Figure 4.**
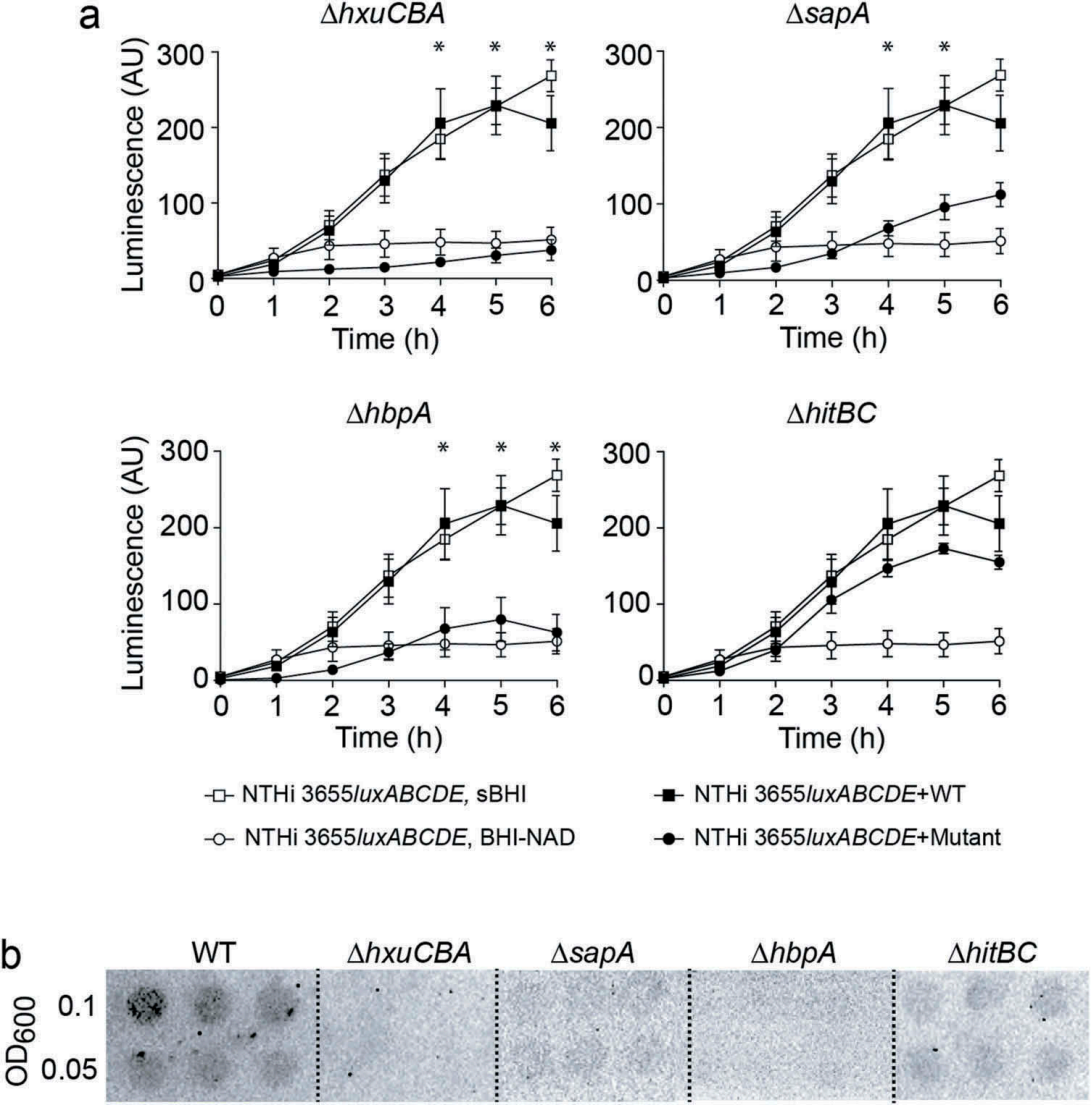
**HxuCBA, SapA and HbpA participate in heme inter-bacterial donation. (a)** Heme-starved NTHi 3655*luxABCDE* was used as a heme-recipient strain. Heme donation allowed recipient´s growth, measured as a means of luminescence. NTHi 3655*luxABCDE* co-culture with Hi RdKW20 WT rendered significant recipient growth, comparable to that of NTHi 3655*luxABCDE* grown in sBHI; in contrast, NTHi 3655*luxABCDE* co-culture with Δ*hxuCBA*, Δ*sapA* or Δ*hbpA*, did not promote recipient strain growth at the same extent (see growth time points 4, 5 and 6 h). HitABC does not contribute to surface-bound heme inter-bacterial donation. NTHi 3655*luxABCDE* co-culture with both Hi RdKW20 WT and ∆*hitBC* rendered significant recipient growth. White symbols, controls showing recipient strain growth in sBHI (square) or BHI-NAD (circle). Black symbols, recipient strain growth when co-cultured with WT (square) or mutant (circle) strains. *Indicates significant differences when comparing luminescence upon recipient co-culture with WT (square) or mutant (circle) strains. Data are shown as luminescence arbitrary units (AU) and represent mean ± SEM values. Statistical comparisons of the means were performed with two-way ANOVA (Bonferroni multiple comparisons test). **(b)** Semi-quantitative measure of heme binding on the surface of Hi RdKW20 WT and mutant strains. Normalized bacterial suspensions were 2-fold serially diluted (OD_600_ = 0.1 and 0.05) and spotted on a PVDF filter. ECL was used for heme detection. Blots are representative images of at least three independent experiments.

PE functions as a heme reservoir at the bacterial surface, which can be donated to other *H. influenzae* bacterial cells starved of heme [63]. We asked if this PE trait could be extended to the other heme-binding proteins. Monitoring heme donation as a means of luminescent emission requires co-culturing a recipient luminescent strain starved of heme and a donor strain cultured in the presence of heme [63]. Unexpectedly, NTHi375 was a poor heme donor, rendering very limited recipient growth (Fig. S6A); in contrast, *H. influenzae* reference strain RdKW20, which also contains the heme acquisition systems under study (*hxuCBA*, HI0262_HI0263_HI0264; *sapABCDFZ*, HI1638_HI1639_HI1640_HI1641_HI1642_HI1643; *hbpA*, HI0853) [97], donates heme at a significantly higher rate (Figure 4(a)). While comparing NTHi375 and RdKW20 donor strains, we found that the *hbpA* gene expression was significantly higher in the latter (Fig. S6B). This limitation by NTHi375 led us to perform gene inactivation in RdKW20 to analyze heme storage and inter-bacterial donation. The growth of the generated mutants in sBHI was comparable to that of the WT strain (Fig. S6C). We assessed the use of heme derived from SapA, HbpA, and HxuCBA by setting up co-cultures of NTHi3655*luxABCDE* starved of heme with RdKW20 WT or mutant strains cultured with heme. NTHi3655*luxABCDE* grew better when incubated with RdKW20 WT as compared to co-culture with RdKW20Δ*sapA*, RdKW20Δ*hbpA,* or RdKW20Δ*hxuCBA* mutants (Figure 4(a)). We next asked if heme interbacterial donation relates to its binding. To do so, we employed the free iron-binding system HitABC, in which HitA is a periplasmic protein that binds free iron linked to the HitBC transporter located at the inner membrane [98–101]. Inactivation of the *hitABC* system was performed in RdKW20 (HI0097_HI0098_HI0099). Growth of the generated mutant in sBHI was comparable to that of the WT strain (Fig. S6C). When co-culturing NTHi3655*luxABCDE* starved of heme with RdKW20Δ*hitBC* cultured with heme, NTHi3655*luxABCDE* growth was comparable to that obtained by co-culturing with the WT strain (Figure 4(a)), excluding participation of this free iron transport system in heme donation [98,99,101]. Lastly, deficiency in heme inter-bacterial donation may relate to lower heme binding at the bacterial surface. In fact, more heme was bound to WT than to the *hxuCBA, sapA,* and *hbpA* mutants. Unexpectedly, RdKW20Δ*hitBC* displayed a slight reduction in heme binding, but to a lower extent than that by the Δ*hxuCBA*, Δ*sapA,* and Δ*hbpA* strains (Figure 4(b)).

Overall, these results suggest that heme binding may work as a storage pool for heme to be shared between *H. influenzae* bacterial cells during shortage. This ability which was previously observed for the outer membrane lipoprotein PE [63] is now extended to HxuCBA, SapA, and HbpA.

## Moonlighting features of H. influenzaeheme-binding systems

**Figure 5.**
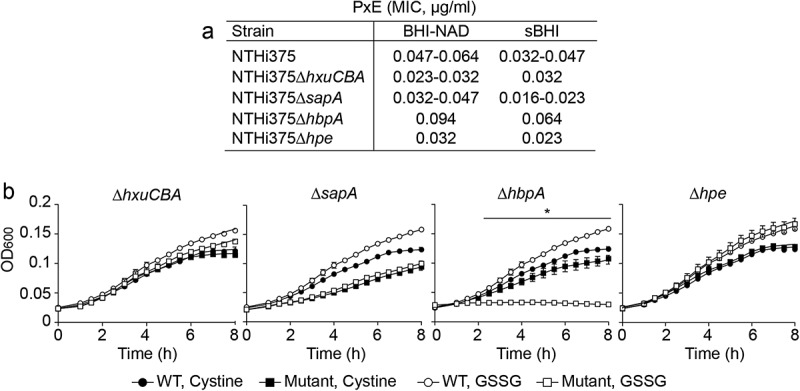
**Moonlighting features of NTHi heme acquisition systems. (a)** HxuCBA, SapABCDFZ and PE are involved in NTHi resistance to PxE. MIC for PxE was shown to be lower for Δ*hxuCBA*, Δ*sapA* and Δ*hpe* mutants than for the WT strain. Inactivation of the *hbpA* gene rendered higher MIC for PxE. Bacterial inocula were prepared on heme-deficient or sBHI media. **(b)** HbpA, but not HxuCBA, SapABCDFZ or PE, participates in glutathione import by NTHi. Bacteria were grown in CDMM supplemented with cystine or GSSG. CDMM+cystine sustained bacterial growth in all cases. NTHi375Δ*hbpA* growth was lower in CDMM+GSSG than in CDMM+cystine (at 2.5 h, p < 0.05; 3 h, p < 0.001; from 3.5 to 8 h p < 0.0001). Data are shown as mean ± SEM. Statistical comparisons of the means were performed using two-way ANOVA (Tukey´s multiple comparisons test).

Besides heme binding, SapA is required for NTHi resistance to antimicrobial peptides (AMP) and potassium acquisition, HbpA is essential for glutathione import, and PE binds extracellular matrix proteins [62,91,102–106]. To get further insight on the multi-functionality of the heme acquisition systems under study, we tested their contribution to NTHi resistance to polymyxin E (PxE) as a representative AMP. Inactivation of the *hxuCBA, sapA,* and *hpe* genes rendered reduced MIC for this AMP; conversely, *hbpA* gene inactivation increased PxE MIC. Mutant strains showed comparable relative phenotypes when grown in heme-deficient medium or in sBHI (Figure 5(a)). Moreover, we tested their contribution to glutathione import, a vital L-cysteine-containing tripeptide mediating protection against oxidative, xenobiotic, and metal iron stresses [107,108]. *H. influenzae* is a cysteine and glutathione auxotroph, and imports both molecules. Exogenous cysteine and glutathione, and their oxidized forms cystine and glutathione disulfide (GSSG), sustain *H. influenzae* growth comparably [73]. NTHi375 WT and mutant strains were inoculated in CDMM supplemented with cystine or GSSG. CDMM supplemented with cystine sustained bacterial growth in all cases. In contrast, CDMM supplemented with GSSG did not sustain NTHi375Δ*hbpA* growth. NTHi375Δ*sapA* growth was lower than that of the WT strain, but comparable in CDMM supplemented with cystine or GSSG (Figure 5(b)).

In conclusion, besides their common role in heme binding, HxuCBA, SapA, and PE also account for AMP resistance, and HbpA accounts for gluthatione import.

## NTHi heme acquisition systems contribute to host airways-pathogen interplay

**Figure 6.**
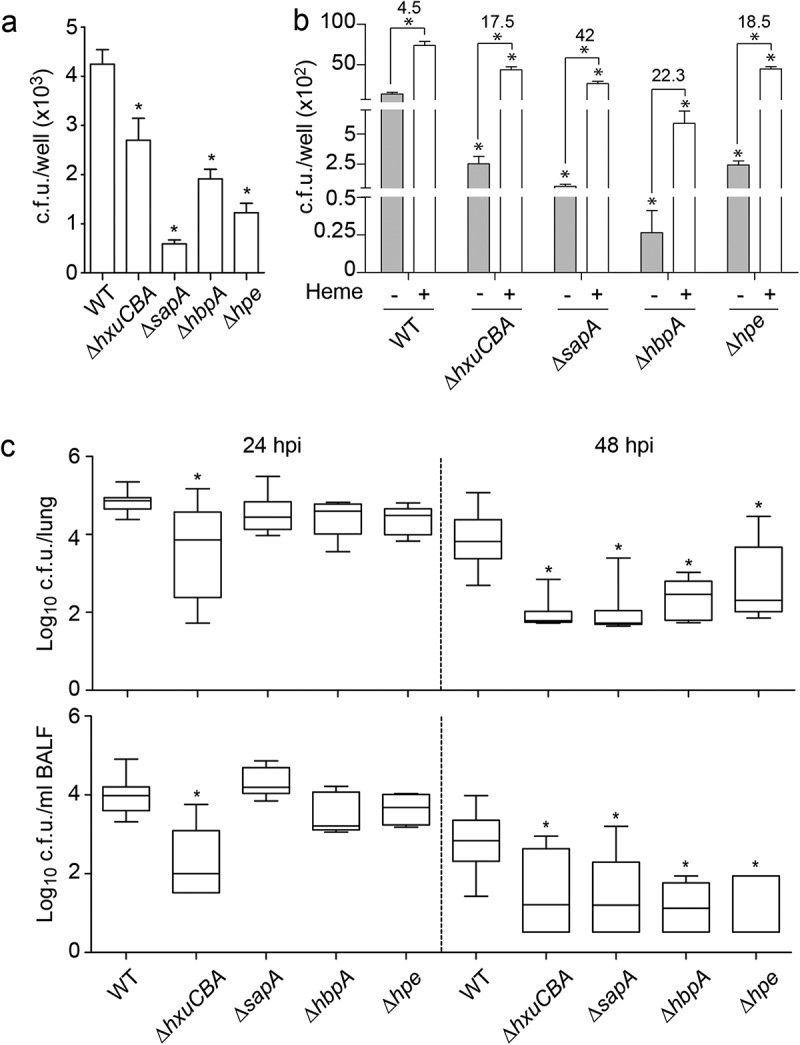
**NTHi heme acquisition systems contribute to the host airway-pathogen interplay. (a)** Effect of heme uptake inactivation in NTHi interplay with cultured cells. A549 cells were used to quantify invasion by NTHi375 WT, Δ*hxuCBA*, Δ*sapA*, Δ*hbpA* and Δ*hpe* strains. Gene inactivation rendered significantly lower entry into A549 cells than that shown by the WT strain (Δ*hxuCBA*, p < 0.05; Δ*sapA* and Δ*hpe*, p < 0.0001; Δ*hbpA*, p < 0.001). Mean ± SEM values are shown, statistical comparisons of the means were performed using one-way ANOVA (Dunnett’s multiple comparison test). **(b)** Inactivation of heme-binding systems lowers NTHi interaction with airway epithelial cells in a heme-dependent manner. BHI-NAD (gray bars) and sBHI (white bars) led to decreased A549 invasion rates for the mutants, compared to that of the WT strain (p < 0.0001). Bacterial growth in sBHI increased invasion rates, compared to BHI-NAD (for WT, Δ*hxuCBA*, Δ*sapA* and Δ*hpe*, p < 0.0001). Top numbers indicate invasion fold increase for each strain upon growth in sBHI compared to BHI-NAD. Mean ± SEM values are shown, statistical comparisons of the means were performed using two-way ANOVA (Sidak’s multiple comparison test). **(c)** Effect of heme uptake inactivation in NTHi pulmonary infection. CD1 mice were intranasally infected with WT and mutant strains, euthanized at 24 and 48 hpi, and bacterial loads quantified. Results are reported as log_10_ CFU/lung and log_10_ CFU/ml BALF in upper and lower panels, respectively, and represented as box plot graphs (lines inside boxes represent median values). Statistical comparisons were performed using one-way ANOVA (Dunnett’s multiple comparison test). In the lungs, NTHi375Δ*hxuCBA* showed significantly lower loads at 24 hpi (Δ*hxuCBA*, p < 0.0005), and all mutants showed significantly lower loads at 48 hpi (Δ*hxuCBA*, Δ*sapA*, p < 0.0001; Δ*hbpA*, p < 0.001; Δ*hpe*, p < 0.05) than those shown by the WT strain. In BALF samples, NTHi375Δ*hxuCBA* showed significantly lower loads at 24 hpi (p < 0.0001), and all mutants showed significantly lower loads at 48 hpi (Δ*hxuCBA*, p < 0.05; Δ*hpe*, p < 0.01; Δ*sapA* and Δ*hbpA* p < 0.005) than those shown by the WT strain.

Production of dedicated heme scavenging machinery may also relate to successful bacterial-host interplay [1,6]. We asked whether inactivation of the systems under analysis alters NTHi interplay with A549 type II human pneumocytes. Epithelial invasion by NTHi375Δ*hxuCBA*, NTHi375Δ*sapA*, NTHi375Δ*hbpA,* and NTHi375Δ*hpe* was lower than that observed for the WT strain (Figure 6(a)). All strains stimulated epithelial IL-8 secretion, but such inflammatory response was lower for NTHi375Δ*hxuCBA*, NTHi375Δ*sapA,* and NTHi375Δ*hpe* than for the WT strain (Fig. S7A). We next assessed if heme availability modulates the NTHi-host cell interplay by growing NTHi375 WT and mutant strains in heme-deficient medium or sBHI before infection. Epithelial invasion for the mutants was lower than for the WT strain in all cases. Moreover, BHI-NAD rendered lower invasion when compared to that of bacteria grown in sBHI. However, while comparing invasion by BHI-NAD to that of sBHI grown bacteria, the ratio of increased invasion upon growth in sBHI was higher for the mutants (for NTHi375Δ*hxuCBA*, 17.5-fold; for NTHi375Δ*sapA*, 42-fold; for NTHi375Δ*hbpA*, 22.3-fold; for NTHi375Δ*hpe*, 18.5-fold) than for the WT strain, 4.5-fold (Figure 6(b)). At a lower extent, similar observations were obtained when assessing epithelial adhesion (Fig. S7B). Last, we used an *in vivo* mouse respiratory infection model system [79–81,85,86]. At 24 hpi, NTHi375Δ*hxuCBA* lung and BALF bacterial numbers were lower than those recovered for the WT strain (Figure 6(c), left panels). At 48 hpi, the four tested mutants rendered numbers lower than those of the WT strain in both lung and BALF samples (Figure 6(c), right panels).

In summary, these results support that, under the conditions tested, inactivation of the HxuCBA, SapABCDFZ, HbpA, and PE systems impairs airway infection by NTHi, and that bacterial heme binding may facilitate its interplay with host epithelial cells.

## Discussion

Microorganisms have evolved or acquired a variety of specialized iron uptake systems to overcome its environmental limitation and take up iron from extracellular sources, including during infection [109]. For many Gram negative bacteria (including uropathogenic *E. coli, Pseudomonas aeruginosa, Neisseria meningitidis, Acinetobacter baumannii*), Gram positive species (*Staphylococcus aureus*), and fungal pathogens (*Candida albicans, Cryptococcus neoformans*), heme provides the potential for a rich source of iron [110]. Here, we tackled the *H. influenzae* case, a human host-restricted opportunistic pathogen colonizing the lower airways of patients who undergo iron homeostasis unbalancing (i.e. COPD), and that requires exogenous heme for aerobic respiration, lacks siderophore production, and presents a panel of heme acquisition systems. In this study, differential contribution of HxuCBA, SapA, HbpA, and PE systems to NTHi respiratory infection was evaluated.

The proteins HxuA, SapA, HbpA, and PE share the ability to bind heme. We provide evidence for heme binding to SapA and HbpA Cluster C substrate-binding proteins (SBP) as well as information on the shared predicted structural basis of such binding. In fact, one striking feature of the Cluster C family is that members play dual roles in nutrient uptake, importing their canonical substrates and heme. In NTHi, four Cluster C proteins with capability to bind heme have been identified: HbpA, SapA, and OppA, which bind hydrophobic oligopeptides, and NTHI0310 with an unknown canonical substrate specificity. These proteins present overlapping functionality and multisubstrate specificity [90]. This may not be exclusive to NTHi given that the *E. coli* Cluster C nickel SBP NikA binds heme and plays a role as a heme chaperone in the periplasm [111]. Regarding HxuA, it should be stressed that our structural analyses may support heme binding, together with the experimental evidence presented in Figure 4 whereas previous data suggests heme binding to HxuC, when expressed together with the NTHi TonB complex in an *E. coli* heterologous host [53]. The mode of heme binding to PE has been predicted previously [63], and our docking analysis supports this and gives further details about the residues involved.

Apart from the requirement for aerobic respiration, bound heme may also be stored at the bacterial surface or donated to neighboring heme-starved bacterial cells, by contribution of these systems. SapA and HbpA are predicted periplasmic proteins, and may need to be coupled to other currently unknown bacterial systems for effective heme uptake and donation. Moreover, we should not exclude that deficiency in heme donation by the *sapA* and *hbpA* mutants could also be an indirect effect. We acknowledge that using RdKW20 mutants to assess heme inter-bacterial donation may be a potential limitation given our use of the NTHi375 strain in the entire study, however, that it should be noted that RdKW20 rendered a useful tool to generate this specific information. Expression of the *hxuA, sapA,* and *hpe* genes was comparable between NTHi375 and RdKW20 WT strains, and was in parallel to expression to the same level of the *hitB, tonB, tbpA,* and *dppB* heme-iron uptake related genes; only *hbpA* gene expression was higher in RdKW20 than in NTHi375 strain, which could have an effect on the observed differences between strain backgrounds (Fig. S6B). Although the precise reason for limited heme inter-bacterial donation by NTHi375 is unknown, we provide several evidences suggesting heme binding at this bacterial surface: heme availability increased alveolar epithelial infection by NTHi375, and also increased NTHi375 susceptibility to PxE (more clearly shown for WT, *sapA, hxuCBA,* and *hpe* mutant strains) supporting the notion of AMP and heme competition for binding to SapA [58], HxuA or PE.

When tackling a possible relationship among heme uptake systems at the gene expression level, a prominent role for HbpA was observed, based on its high expression and on the effect of its inactivation on the expression of the *hxuA, sapA,* and *hpe* genes, suggesting HbpA contribution to down-regulating the activity of other heme piracy systems. However, HbpA does not seem to participate in heme sensing. In fact, in NTHi375 WT, Δ*hbpA,* and Δ*hpe* strains, expression of the *hxuA, sapA,* and *hpe* genes was higher in heme-restricted than in sBHI medium, supporting the notion that the expression of those genes is up-regulated upon heme restriction in a HbpA- and PE-independent manner. Conversely, in NTHi375 WT, Δ*sapA,* and Δ*hpe* strains, expression of the *hbpA* gene was lower upon heme depletion, suggesting *hbpA* gene expression being down-regulated upon heme restriction in a SapABCDFZ- and PE-independent manner. For unknown reasons, we noticed that expression of the *hxuA, sapA,* and *hpe* genes in NTHi375Δ*hbpA* was higher when conventionally grown in sBHI (Figure 3(a)) than in sBHI while assessing the effect of heme availability (Figure 3(b)). We acknowledge that BHI is a medium with non-defined composition that could render reproducibility issues, and emphasize the importance of preserving the use of exactly the same product in each experimental design, as performed in this study. Although to a lesser extent for HxuCBA, SapABCDFZ, and PE, heme acquisition interlinks may contribute to regulating the expression of the different systems, altogether compatible with the notion of a coordinated heme uptake network preventing toxicity by heme excess.

**Figure 7.**
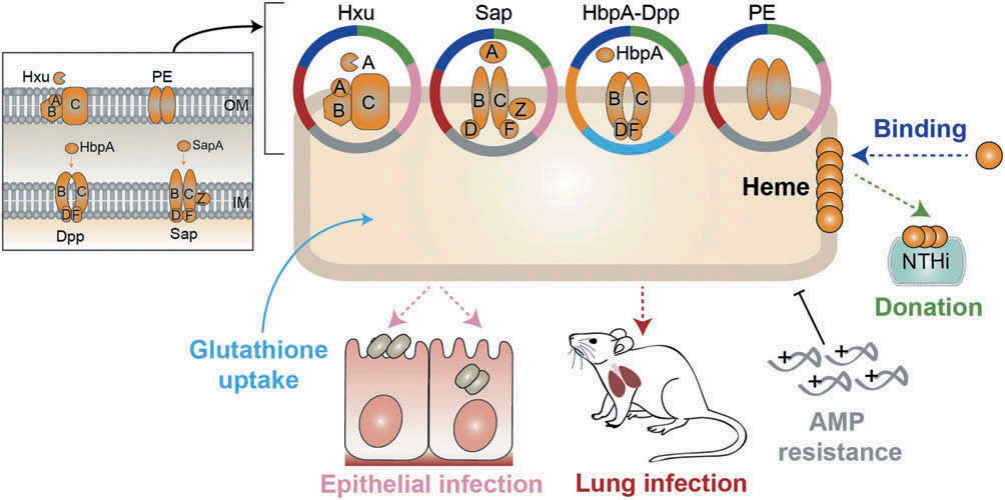
Schematic model showing moonlighting patterns by HxuCBA, SapABCDFZ, HbpA-DppBCDF and PE heme acquisition systems.

Notably, the heme uptake systems studied present moonlighting features (summarized in Figure 7). We highlight a relationship between NTHi heme acquisition and AMPs. In addition to the already reported involvement of SapABCDFZ in resistance to AMPs [102,103,112], HxuCBA and PE also seem to subvert these elements of the human soluble innate immunity. This was not the case for NTHi375Δ*hbpA*, which could be linked to the observed higher expression levels of the *hxuA, sapA,* and *hpe* genes upon *hbpA* gene inactivation. Monitoring AMP resistance was motivated by an earlier study where we conducted a genomic analysis of three NTHi clonal isolates serially collected from a bronchiectasis patient, which showed increasing AMP resistance and carried amino acid substitutions in SapABCDFZ and HxuCBA [113]. Based on that and current observations, molecular crosstalk between NTHi heme acquisition and AMP resistance will be addressed in further studies. We also asked if glutathione import could add to protein multi-functionality. This was only the case for HbpA, in line with previous observations [62].

The use of cultured airway epithelia and murine pulmonary infection model systems revealed a significant involvement for heme binding systems in the bacterial-host interplay. Data obtained for NTHi375Δ*hpe* support the role of PE as an adhesin [105,106]. In contrast, the *sapA* gene inactivation has been shown to increase bacterial invasiveness [104], which could relate to the use of non-polarized (this study) *versus* polarized epithelial cells grown at an air-liquid interface [104]. On the other hand, increased invasion by the WT NTHi strain when pre-grown in sBHI, and heme rescue of *hxuCBA, sapA, hbpA,* and *hpe* mutant deficiencies upon epithelial infection, could relate to pathogen subversion of host cell heme receptors such as heme uptake proteins HCP1 and HRG1 by using bound heme as a ligand. Our results somehow differ from those shown by transient iron deprived bacteria developing intracellular bacterial communities [64,114], which could relate to the use of different host cell lines, bacterial strains, or growth conditions. A role for HbpA and HxuCBA in causing bacteremia has been shown in a rat model of invasive disease [55,60], and for SapABCDFZ in a chinchilla model of otitis media (SapA, SapF [103,112,115]) and a mouse model of lung infection (SapF [116]). By using a mouse model of lung infection, we expanded previous observations on HbpA, HxuCBA and SapABCDFZ, and provided new roles for the PE heme-acquisition system *in vivo*.

In conclusion, the “iron-loving” bacterial species *H. influenzae* has evolved redundant mechanisms to obtain heme from the human host, and several of them present specific moonlighting properties. We also began elucidating the complex regulatory networks used by NTHi to adapt to the host airways. In general terms, pharmacological modification of host heme-iron metabolism and/or acquisition may hold a key to develop novel strategies to treat or prevent infection. Iron chelators with improved pharmacokinetic properties may become important antimicrobials, and heme-iron transporters and acquisition pathways attractive targets for the development of new treatments to fight infections in an era of increasing burden of antibiotic resistance.
