# Supplementary material for: Moonlighting of Haemophilus influenzae heme acquisition systems contributes to the host airway-pathogen interplay in a coordinated manner
Source: Virulence. 2019 Apr 11;10(1):315–33. doi: 10.1080/21505594.2019.1596506 (PMC6550540; doi:10.1080/21505594.2019.1596506)
Supplement: Supplemental Material [file kvir-10-01-1596506-s001.pdf]

Supplementary Material

Moonlighting *Haemophilus influenzae* heme acquisition systems contributes to the host airway-pathogen interplay in a coordinated manner

Irene Rodríguez-Arce[1+], Tamim Al Jubair[2,3+], Begoña Euba[1,4+], Ariadna Fernández-Calvet[1], Celia Gil-Campillo[1], Sara Martí[4,5], Susanna Törnroth-Horsefield[6], Kristian Riesbeck[2], Junkal Garmendia[1,4*]

[1]Instituto de Agrobiotecnología, CSIC-Gobierno Navarra, Mutilva, Spain; [2]Clinical Microbiology, Department of Translational Medicine, Faculty of Medicine, Lund University, Malmö, Sweden; [3]Department of Biomedical Sciences, Faculty of Health Sciences, University of Copenhagen, Copenhagen, Denmark; [4]Centro de Investigación Biomédica en Red de Enfermedades Respiratorias (CIBERES), Madrid, Spain; [5]Departamento Microbiología, Hospital Universitari Bellvitge, University of Barcelona, IDIBELL, Barcelona, Spain; [6]Department of Biochemistry and Structural Biology, Center for Molecular Protein Science, Lund University, Lund, Sweden

[+]These authors contributed equally to this work.

Content:

Figure S1. Supportive information on approaches employed for gene inactivation.

Figure S2. Sequence alignment of *Hp*HbpA, *Hi*HbpA, *Hi*SapA and *Ec*DppA.

Figure S3. Comparison of ligand binding site in *Hi*HbpA, *Hi*SapA, *Hp*HbpA and *Ec*DppA.

Figure S4. Structural prediction of heme binding to PE and HxuA.

Figure S5. Effects of the inactivation of heme uptake systems in *H. influenzae* growth.

Figure S6. Experimental support for the use of RdKW20 strain in heme donation assays.

Figure S7. Effects of heme uptake gene inactivation on epithelial cell infection by NTHi.

Table S1. Plasmids used in this study.

Table S2. Chemically defined minimal medium (CDMM): composition and preparation.

Table S3. Primers used in this study.

Table S4. HADDOCK docking statistics.

Table S5. Minimal inhibitory concentration of 16 antibiotics against NTHi WT and mutant strains determined by microdilution.

Supplementary Figures

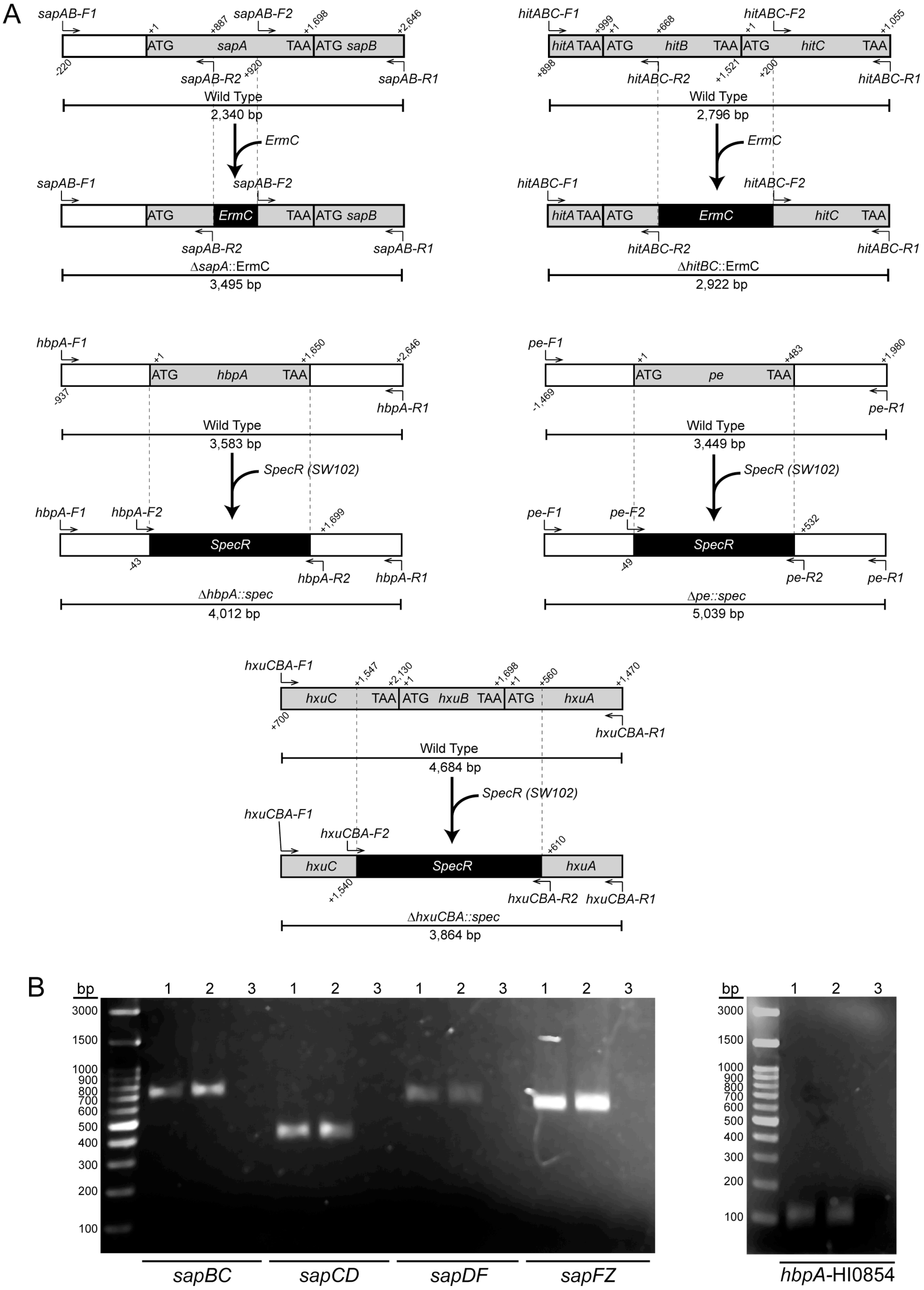

Figure S1. Supportive information on approaches employed for gene inactivation. (A) Design of gene disruption cassettes. Detailed representation of gene inactivation cassettes used to disrupt the *hxuCBA*, *sapA*, *hbpA*, *hpe* and *hitBC* genes in NTHi strains. **(B)** The *sap* gene cluster contains 6 genes (*sapA* to -*Z*) [1]. The *hbpA*-HI0854 gene

cluster contains 2 genes (*hbpA*-Hi0854) [2]. RNA was prepared from mid-log-phase sBHI broth cultures, and the mRNA was reversely transcribed. Primers spanning the 6 and 2 open reading frames, respectively, were used to amplify cDNAs in the presence (+) or absence (-) of RT. PCR products were separated by gel electrophoresis. PCR products size: *sapBC*, primer pair 1842+1843, 689 bp; *sapCD*, primer pair 1861+1862, 395 bp; *sapDF*, primer pair, 1863+1864, 634 bp; *sapFZ*, primer pair 1848+1849, 605 bp; HI0854, primer pair 1869+1870, 94 bp. 1, NTHi375 WT cDNA; 2, NTHiΔ*sapA* / NTHiΔ*hbpA* cDNA; 3, negative control, mutant RNA without RT.

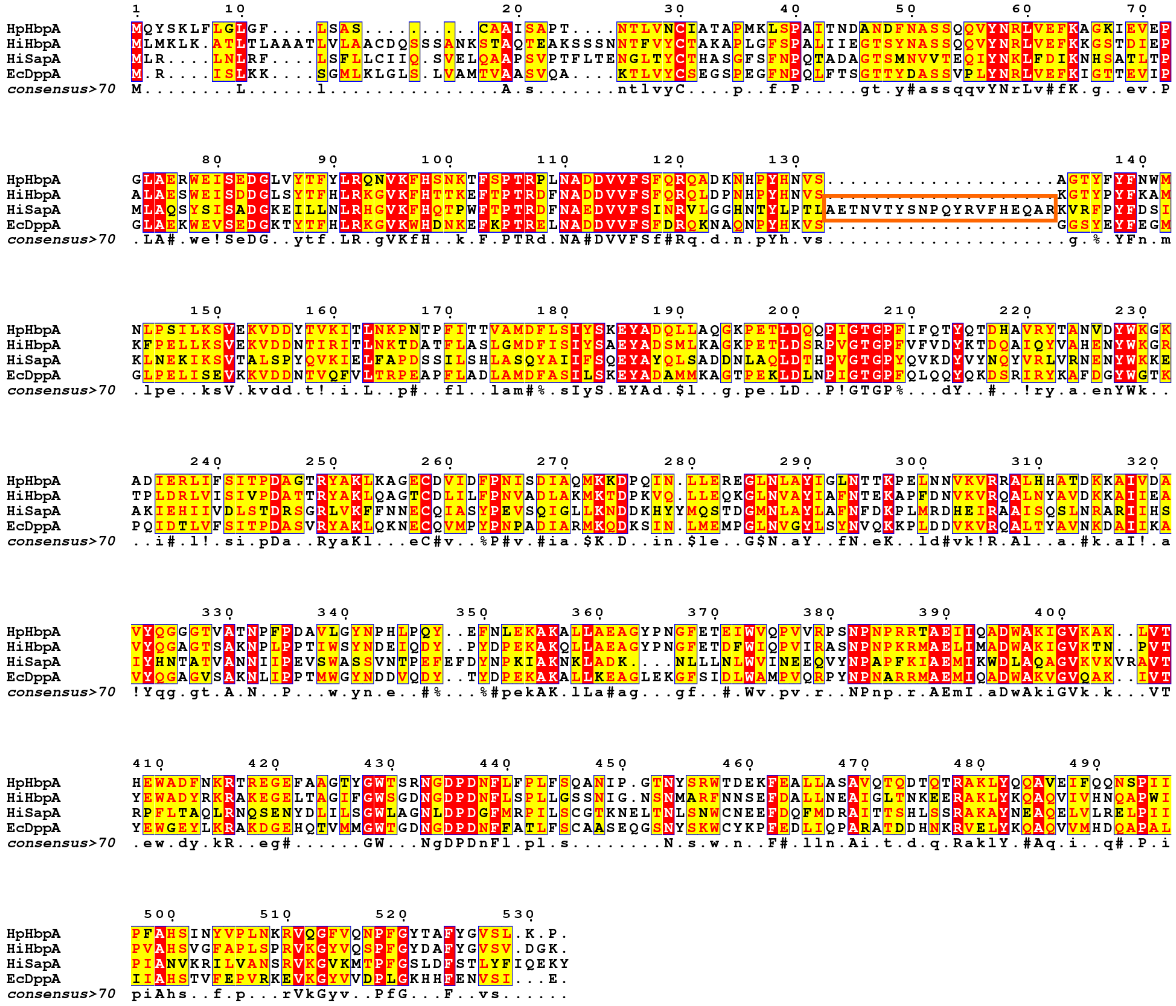

Figure S2. Sequence alignment of *Hp*HbpA, *Hi*HbpA, *Hi*SapA and *Ec*DppA. Sequences were aligned using the T-Coffee server (http://tcoffee.crg.cat) with the Expresso option [3], and the alignment was visualized using ESPript 3.0 [4]. Strictly conserved residues are highlighted in red and residues with a similarity score >0.7 are highlighted in yellow.

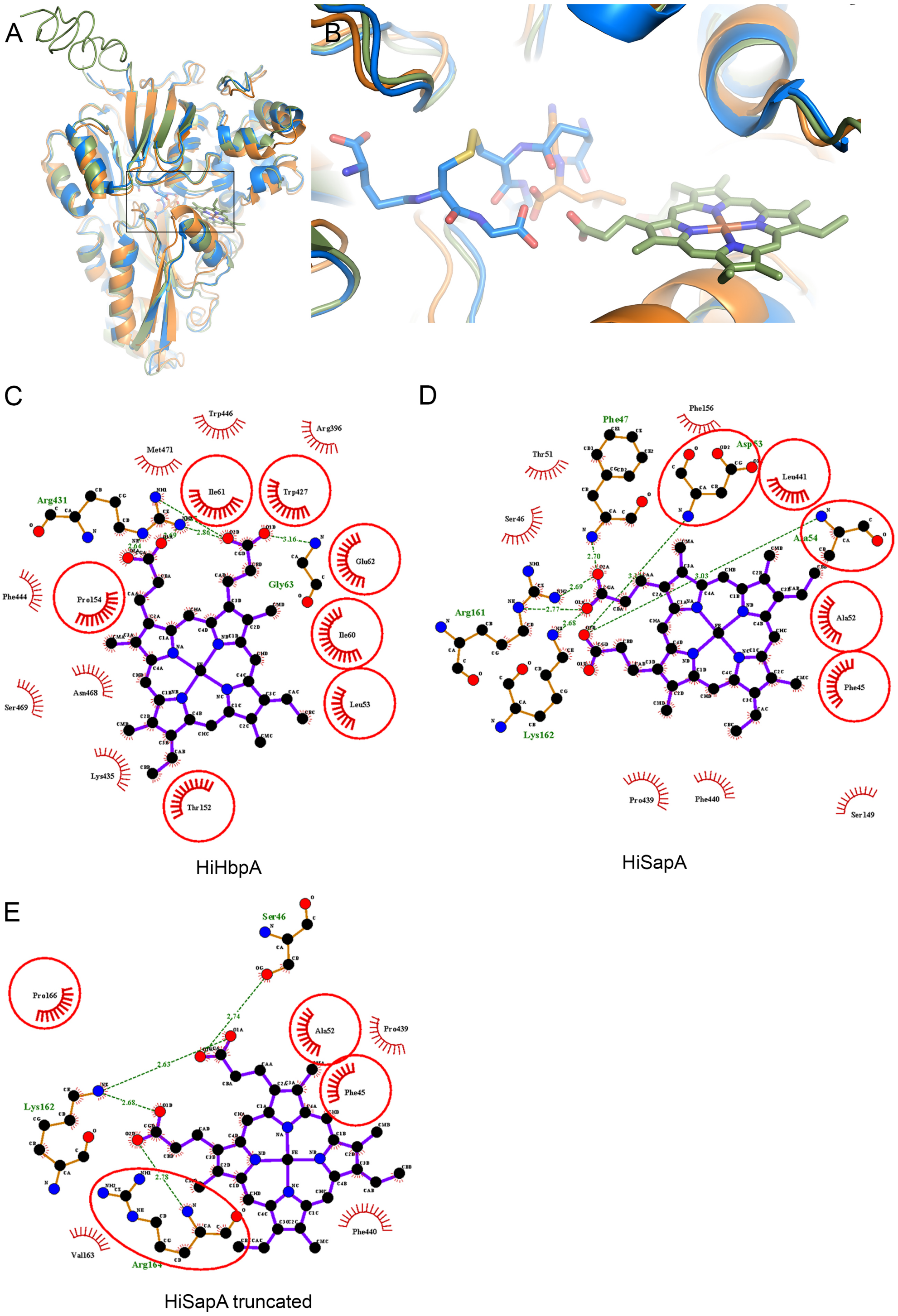

Figure S3. Comparison of ligand binding site in *Hi*HbpA, *Hi*SapA, *Hp*HbpA and *Ec*DppA. (A) Overlay of *Hi*HbpA homology model with docked heme (top docking solution, green), *Hp*HbpA with bound glutathione (blue, PDB code 3M8U) and *Ec*DppA with bound dipeptide (orange, PDB code 1DPP). All ligands bind in a similar location, bridging the N- and C-terminal domains. **(B)** Zoom-in of the ligand binding sites in A showing that the predicted heme binding site in *Hi*HbpA is distinct from the peptide binding sites in *Hp*HbpA and *Ec*DppA. **(C)-(E)** LigPlot^{+} analysis of top heme docking solution for *Hi*HbpA, *Hi*SapA and truncated *Hi*SapA. Residues common for all three binding sites are marked with red circles. Hydrogen bonds are shown as green dotted lines. PyMol versions of the same plots can be found in **Fig. 2** in the main text with common residues coloured yellow.

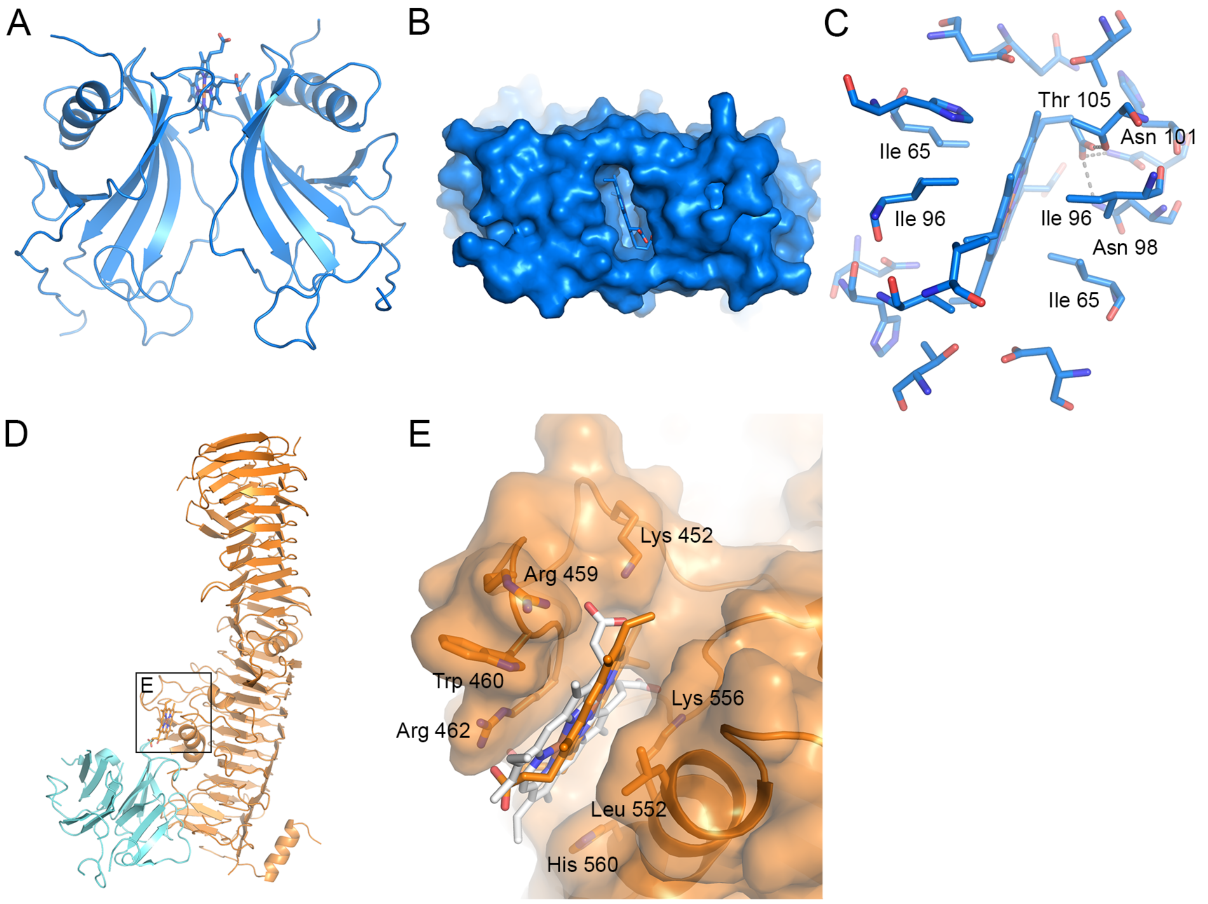

Figure S4. Structural prediction of heme binding to PE and HxuA. HADDOCK was used to dock heme B to the crystal structures of PE (PDB code 3ZH5) and HxuA (PDB code 4RM6). **(A)** Cartoon representation of the top scoring docking solution for PE. **(B)** Same as in (A), shown in surface representation and seen from the top. **(C)** LigPlot^{+} analysis of the predicted heme binding site with binding site residues shown in a stick representation, and predicted hydrogen bonds are shown as dotted lines. **(D)** Top scoring docking solution for HxuA (yellow). Hemopexin (cyan) taken from the crystal structure of the HxuA-hemopexin complex (PDB code 4RT6) is shown for reference but was not included in the docking experiment. **(E)** Zoom-in on the predicted heme binding site showing the two best scoring heme docking solutions (yellow and white). Residues lining the pocket are shown in stick representation.

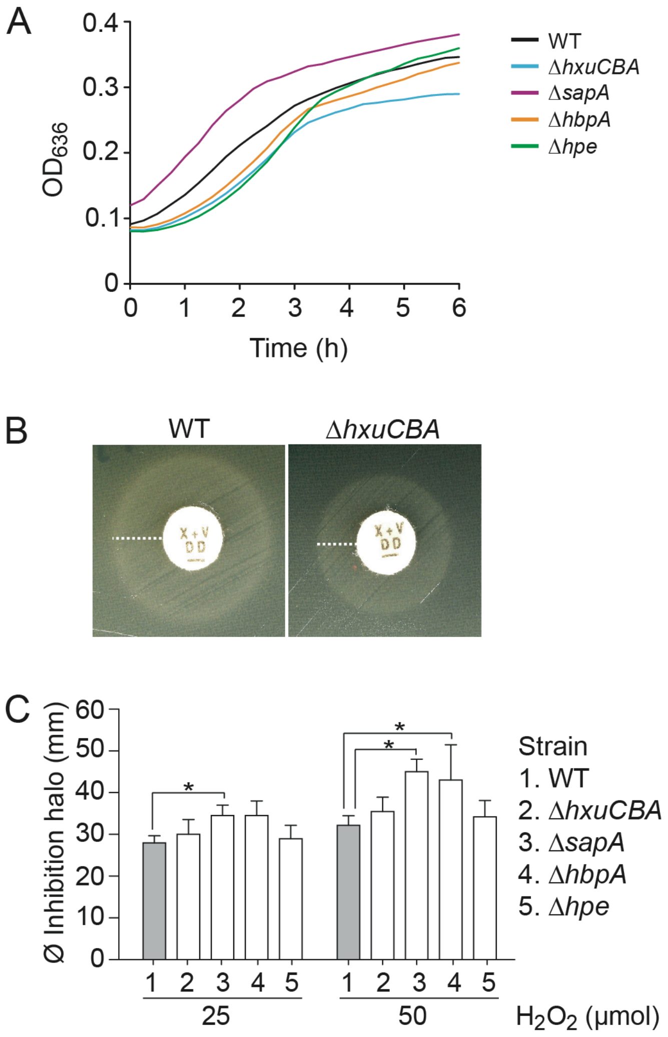

Figure S5. Effects of the inactivation of heme uptake systems in *H. influenzae* growth. **(A)** Growth in sBHI is shown for NTHi375 WT and mutant strains, as a mean of OD_{636} at the indicated time points. **(B)** Bacterial growth on BHI agar around sterile X+V factor disks is shown for NTHi375 WT and Δ*hxuCBA* strains. A representative image per strain is shown. **(C)** Bacterial growth inhibition on sHTM agar around sterile paper disks soaked with H_2O_2. Increased growth inhibition was recorded by Δ*sapA* and Δ*hbpA* mutants and compared to the WT strain (with 25 µmol H_2O_2, Δ*sapA*, $p<0.05$; with 50 µmol H_2O_2, $p<0.0001$ and $p<0.005$, respectively). Means ± SD of three independent experiments expressed as diameter of growth inhibition halo (mm) are shown. Statistical comparisons of the means were performed using two-way ANOVA (Sidak's multiple comparison test).

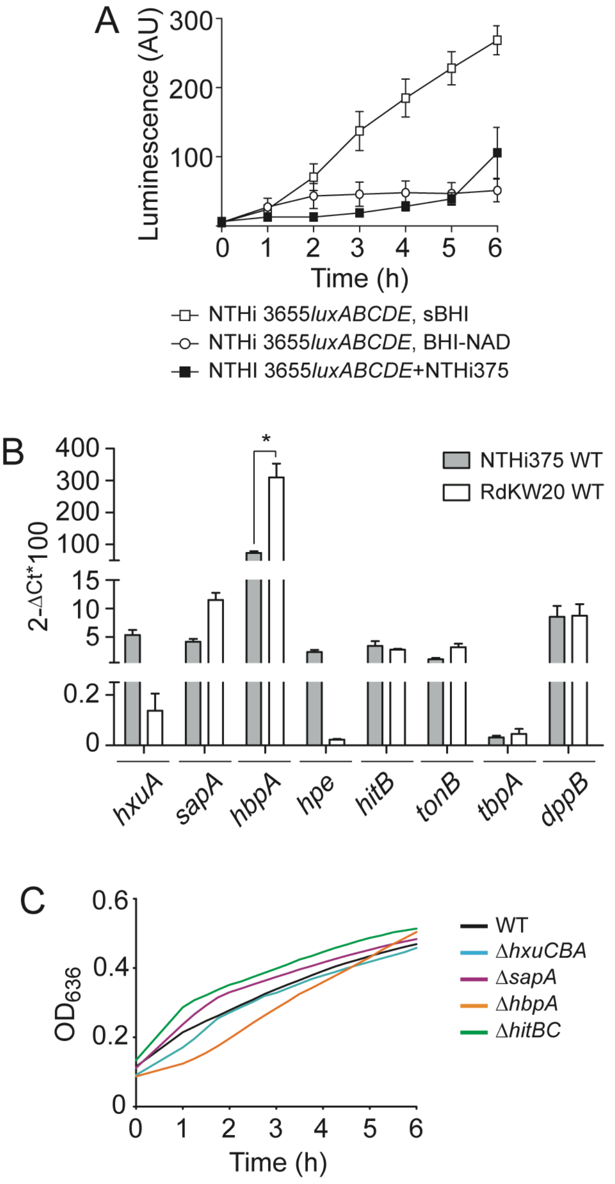

Figure S6. Experimental support for the use of RdKW20 strain in heme donation assays. **(A)** Heme-starved NTHi 3655*luxABCDE* co-culture with NTHi375 WT did not render recipient growth. White symbols, controls showing recipient strain growth in sBHI (square) or BHI-NAD (circle). Black symbols, recipient strain growth when co-cultured with NTHi375. Data are shown as mean ± SEM. **(B)** Expression of heme-iron related genes in NTHi375 and RdKW20 WT strains. sBHI grown bacterial cultures were used to quantify expression of the *hxuA*, *sapA*, *hbpA*, *hpe*, *hitB*, *tonB*, *tbpA* and *dppB* genes. The *hbpA* gene expression differed between strains, being higher in RdKW20 than in NTHi375 ($p<0.0001$). Data are shown as mean ± SEM. Statistical comparison of the means performed using two-way ANOVA (Sidak's multiple comparison test). **(C)** Growth in sBHI is shown for RdKW20 WT and mutant strains, as a mean of OD_{636} at the indicated time points.

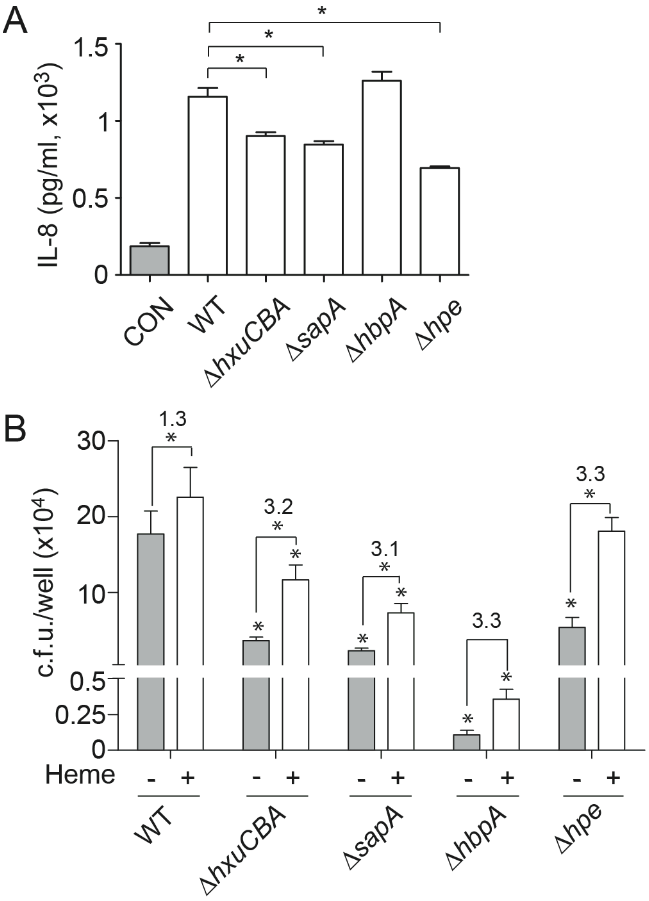

Figure S7. Effects of heme uptake gene inactivation on epithelial cell infection by NTHi. **(A)** IL-8 release was lower in cells infected by NTHi375Δ*hxuCBA*, Δ*sapA* and Δ*hpe* mutants than by the WT strain (Δ*hxuCBA*, p<0.05; Δ*sapA*, p<0.05; Δ*hpe*, p<0.0001). Data are expressed as pg/ml. Means ± SEM are shown, statistical comparisons of the means were performed using one-way ANOVA (Dunnett's multiple comparison test). **(B)** Inactivation of heme-binding systems results in a decreased NTHi adhesion to airway epithelial cells. A549 cells were used to quantify cell adhesion by NTHi375 WT and mutant strains. BHI-NAD (gray bars) and sBHI (white bars) led to decreased A549 adhesion rates for the mutants, compared to that of the WT strain (p<0.0001, except for NTHi375Δ*hpe* when grown in sBHI). Bacterial growth in sBHI increased adhesion compared to BHI-NAD (for WT, p<0.05; Δ*hxuCBA*, p=0.0001; Δ*sapA*, p<0.05; Δ*hpe*, p<0.0001). Top numbers indicate fold increased adhesion for each strain upon growth in sBHI compared to BHI-NAD. Data are expressed as CFU/well. Means ± SEM are shown, statistical comparisons of the means were performed using two-way ANOVA (Sidak's multiple comparison test).

Table S1. Plasmids used in this study.

**Plasmids**	**Description**	**Source**
pJET1.2/blunt	Cloning vector	Thermofisher Scientific
pGEMT-easy	Cloning vector	Promega
pJET1.2-*hxuCBA*	pJET1.2 derivative containing a 4,684 bp DNA fragment carrying the *hxuC* (1,430 bp, nt 701-2,130), *hxuB* (1,698 bp, nt 1-1698), *hxuA* (1,470 bp, nt 1-1,470) genes and their intergenic regions (75 and 11 bp, respectively)	This study
pJET1.2-*hxuCBA::spec*	pJET1.2-*hxuCBA* derivative containing a 3,864 bp DNA fragment carrying a *hxuCBA::spec* disruption cassette	This study
pGEMT-*sapAB*	pGEMT-easy derivative containing a 2,340 bp DNA fragment carrying the *sapA* (1,698 bp, nt 1-1698) gene, *sapB* (423 bp, nt 1-423) gene and the *sapA* upstream (220 bp) flanking region	This study
pGEMT-*sapA::ermC*	pGEMT-*sapAB* derivative containing a 3,495 bp insert encompassing a *sapA::ermC* disruption cassette	This study
pJET1.2-*hbpA*	pJET1.2 derivative containing a 3,583 bp DNA fragment carrying the *hbpA* gene (1,650 bp), upstream (937 bp) and downstream (996 bp) flanking regions	This study
pJET1.2-*hbpA::spec*	pJET1.2-*hbpA* derivative containing a 4,012 bp DNA fragment carrying a *hbpA::spec* disruption cassette	This study
pJET1.2-*hitABC*	pJET1.2 derivative containing a 2,796 bp DNA fragment carrying the 3´end of *hitA* (102 bp, nt 898-999), *hitB* (1,52 and *hitC* (1,055 bp) genes, and the *hitAB* intergenic region (117 bp)	This study
pJET1.2-*hitBC::ermC*	pJET1.2-*hitABC* derivative containing a 2,922 bp insert carrying a *hitBC::ermC* disruption cassette	This study
pJET1.2-*hpe*	pJET1.2 derivative containing a 3,449 bp DNA fragment carrying the *pe* gene (483 bp), upstream (1,400 bp) and downstream (1,497 bp) flanking regions	This study
pJET1.2-*hpe::spec*	pJET1.2-*pe* derivative containing a 5,039 bp DNA fragment carrying a *pe::spec* disruption cassette	This study
pSBLerm	Source of an Erm^R cassette	[5]
pRSM2832	pKD13 derivative carrying a cassette containing a Spec resistance gene flanked by FRT sites	[6]

Table S2. Chemically defined minimal medium (CDMM): composition and preparation.

**Stock solution 1 (Final volume: 1000 ml)**	
L-Aspartic Acid	5 g
L-Glutamic Acid	13 g
NaCl	58 g
K_2SO_4	10 g
$MgCl_2$	2 g
$CaCl_2$	0,222 g
Ethylenediaminetetraacetate	0,037 g
NH_4Cl	2,2 g
dH_2O	800 ml
Ajust pH to 7.2 with 11,8 ml of 10 N NaOH. Add dH_2O to 1000 ml.	
Autoclave and store at 4ºC	
**Stock solution 2 (2.1+2.1+2.3) (1:1:1)**	
**Solution 2.1 (Final volume: 100 ml)**	
L-Arginine	1,5 g
Glycine	0,15 g
L-Lysine	0,25 g
L-Methionine	0.5 g
L-Serine	0,5 g
Add 0,1N HCl to make 100 ml. Sterilize by filtration and store at 4ºC	
**Solution 2.2 (Final volume: 100 ml)**	
L-Leucine	1,5 g
Dissolve in 100 ml of 0,1N HCl. Sterilize by filtration and store at 4ºC.	
**Solution 2.3 (Final volume: 100 ml)**	
L-Tyrosine	1 g
Dissolve in 20 ml of 1N HCl and add immediately dH_2O to 100 ml.	
Sterilize by filtration and store at 4ºC	
**Stock solution 3 (Final volume: 5 ml)**	
Tween-80	50 µl (0.1% v/v)
Polyvinyl alcohol	5 mg (0.1% w/v)
Sodium lactate	200 mg
Glycerol	0.75 ml (15% v/v)
Add dH_2O to 5 ml. Sterilize by filtration and store at 4ºC	
**Stock solution 4 (Final volume: 100 ml)**	
Uracil	0,2 g
Hypoxanthine	0.04 g
Dissolve in 100 ml of 0,1N HCl. Sterilize by filtration and store at 4ºC	
**Stock solution 5 (Final volume: 100 ml)**	
Inosine	1 g
K_2HPO_4	1,74 g
KH_2PO_4	1,36 g
Add dH_2O to 100 ml. Sterilize by filtration and store at 4ºC	
**Stock solution 6 (Final volume: 100 ml)**	
Hemin	0,1 g
L-Histidine	0,1 g
2,2',2''-nitrilotriethanol	4 ml (4% v/v)
Add dH_2O to 100 ml. Sterilize by heating up to 70ºC for 10 min.	
Store at 4ºC (only 2-3 weeks)	
**Stock solution 7 (Final volume: 10 ml)**	
NAD	0,1 g
Thiamine	0,1 g
Calcium pantothenate	0,1 g
Add dH_2O to 100 ml. Sterilize by filtration and store at -20ºC	
**CDMM Preparation**	
1. Mix to 100 ml final volume of CDMM (upon use)	
Solution 1	10 ml

Solution 2 (2.1+2.2+2.3) (1:1:1)	6 ml
Solution 3	2 ml
Solution 4	5 ml
Add 2,6 ml of 1N NaOH and 52,4 ml of dH_2O	
Solution 5	20 ml
Solution 6	1 ml
Solution 7	0,4 ml
Ajust pH to 7.0	

2. When necessary, add 50 μM cystine or 50 μM GSSG

Table S3. Primers used in this study.

Primer name	Primer ID	Sequence (5'-3')	Purpose	Reference
hxuCBA-F1	1280	AATTATCCCACCGCGAAACTCGTT	Gene inactivation	This study
hxuCBA-R1	1281	GTTATCGTAGTCGCTTTCATTGAC	Gene inactivation	This study
hxuCBA-F2	1282	CCGCAAATCTACATTTTGATAGCCTGTTTAAACAAG GCGATAAATTCATTCCGGGGATCCGTCGACC	Gene inactivation	This study
hxuCBA-R2	1283	AGGATAAATATACTTTGCCATTTGTAACGTTAATTT CCTCTTTATTGATATGTAGGCTGGAGCTGCTTCG	Gene inactivation	This study
sapAB-F1	469	AATGTTGATTAATGGAGAGAAGTTGATT	Gene inactivation	This study
sapAB-R1	470	TTCCCAATGTGAGAGCGCGGCAACATA	Gene inactivation	This study
sapAB-F2	471	TTATTATATGCAATCTACTGATGGTAT	Gene inactivation	This study
sapAB-R2	472	CTTACTTCAGGATAAGAGGCGATTTGA	Gene inactivation	This study
hbpA-F1	1499	GTTTTTTACTCTTTGCATTGGGGTTAGT	Gene inactivation	This study
hbpA-R1	1500	AAACAAAAGGGGTGAGTATAAATTTACA	Gene inactivation	This study
hbpA-F2	1503	TTTAACCATAAATGGTAGGGCATTCCCTGCCTAACA TTGAGGTATGCTTATGATTCCGGGGATCCGTCGACC	Gene inactivation	This study
hbpA-R2	1504	ATAAAAAGCCCTTATAAATAAGGGCTTAAAATAAGT AAATCAATATGAATTATGTAGGCTGGAGCTGCTTCG	Gene inactivation	This study
hitABC-F1	1373	CTTGAACCTTATGAAAAATTAGAAGCACC	Gene inactivation	This study
hitABC-R2	1374	CGGGGTACCACGGCGGATAATAATGCAGCAG	Gene inactivation	This study
hitABC-F2	1375	CGGGGTACCGCGGTTAATTTTGGCGAGAATT	Gene inactivation	This study
hitABC-R1	1278	TAAGCGTAAAAAAGCCCTTTTCTA	Gene inactivation	This study
hpe-F1	1501	CGTGTTGATGACATCGCTTGTGACTTA	Gene inactivation	This study
hpe-R1	1502	AGCGACTGCTTCATATTCGCGCGTAAT	Gene inactivation	This study
hpe-F2	1505	CACACTGTAAAATTCTAGAATAAATGTCAGCTAACA TAAGGAGTAAATAATGATTCCGGGGATCCGTCGACC	Gene inactivation	This study
hpe-R2	1506	GCACCCTAAAAATTGATTAAAATTTAAACTAATTAA GGTGCAGATTTTTTTATGTAGGCTGGAGCTGCTTCG	Gene inactivation	This study
pSBLerm_up	1306	ATAAAGAGGGTTATAATGAACGAG	Gene inactivation	[7]
pSBLerm_down	1307	GGTACACGAAAAACAAGTTAAGGG	Gene inactivation	[7]
*tbpA*qPCR-F3	1471	AAGTAAGAGATCGTAAAGATAATGAAGTAACTG	qRT-PCR	[8]
*tbpA*qPCR-R3	1472	ACCGCGACCTTGTTCTACAACT	qRT-PCR	[8]
*hxuA*qPCR-F2	1661	ACCCAAGGAGCAGAAATAAATG	qRT-PCR	This study
*hxuA*qPCR-R2	1662	TCGCCATTAAGTACCACGAA	qRT-PCR	This study
*sapA*qPCR-F2	1621	GATTCTATCTGGTTGGTTAGCTGGTAA	qRT-PCR	This study
*sapA*qPCR-R2	1622	AGGTGGTAATGGCACGATCC	qRT-PCR	This study
*hbpA*qPCR-F2	1613	CTGGATCGTACGGATAATCTTGG	qRT-PCR	This study
*hbpA*qPCR-R2	1614	AAGGCGCAGGAATATCAGCTAA	qRT-PCR	This study
*hitB*qPCR-F3	1631	AAGTGCGGGTGATTTTTCAG	qRT-PCR	This study
*hitB*qPCR-R3	1630	CAAACTAATGGCAAGGCACA	qRT-PCR	This study
*hpe*qPCR-F2	1609	ATTATCACTTGGGTTACTTACTGCCTG	qRT-PCR	This study
*hpe*qPCR-R2	1610	ACGTATATATCCGCTTCGTACATCAG	qRT-PCR	This study
*dppB*qPCR-F	1422	CACAAGGTGGGCGTTTAGAT	qRT-PCR	This study
*dppB*qPCR-R	1423	GCGTAATAATGGCAAGAGGA	qRT-PCR	This study
*tonB*qPCR-F	1414	GCTACCAAAAGGCGATGAAA	qRT-PCR	This study
*tonB*qPCR-R	1415	TCCGTTCCACTTCCTGCTAC	qRT-PCR	This study
16srRNA-F1	1074	GGCGTTGATGACCGTGAAAC	qRT-PCR	[7]
16srRNA-R1	1075	GCCAGTAATAATCGCCCTCTTCTAG	qRT-PCR	[7]
sapBC-rtPCR-F	1842	GGGTTGGTCAAAATGGAAAA	RT-PCR	This study
sapBC-rtPCR-R	1843	AATATGCCCGACAAAACGAG	RT-PCR	This study
sapCD-rtPCR-F2	1861	GCACAACGACCTACACCAGA	RT-PCR	This study
sapCD-rtPCR-R2	1862	TCGACATCGTGAAAACGAAA	RT-PCR	This study
sapDF-rtPCR-F2	1863	ACAGAAGGCGAAGGACAAAA	RT-PCR	This study
sapDF-rtPCR-R2	1864	CCAAATAAACTGGCGTGACC	RT-PCR	This study
sapFZ-rtPCR-F	1848	CGTAATGGATGAAGGAAAAATGA	RT-PCR	This study
sapFZ-rtPCR-R	1849	GCTTTGGTAACAGGCTCTGC	RT-PCR	This study
HI0854-rtPCR-F	1869	CAGGCCGTTTTGTAAGAGGTT	RT-PCR	This study
HI0854-rtPCR-R	1870	TTTTCGTGTGGATTGCCTTT	RT-PCR	This study

Table S4. HADDOCK docking statistics. Detailed docking statistics for the four top solutions clusters. For PE, only two clusters were obtained. All energies are given in kcal/mol. RMSD represents RMSD of the cluster from the overall lowest-energy structure.

***H. influenzae* HbpA**

	Cluster 1	Cluster 2	Cluster 3	Cluster 4
HADDOCK score	164.2±1.6	157.8±2.8	153.7±9.7	151.7±10.4
Cluster size	36	90	12	26
RMSD (Å)	0.5±0	0.5±0	0.5±0	0.4±0.2
Van der Waals energy	-39.5±5.1	-33.6±4.4	-40.6±11.5	-46.4±3.9
Electrostatic energy	-197.8±10.7	-216.4±25	-213.7±38.8	-106.3±59.0
Desolvation energy	-91.9±5.8	-89.7±7.9	-82.6±6.7	-88.9±5.0
Restraints violation energy	68.0±26.3	88.3±25.8	122.6±36.9	48.4±18.0
Buried surface area (Å^2)	1081±88.6	1029.3±64.9	1075.2±89.8	973.3±17.8
Z-score	-1.1	-0.8	-0.6	-0.5

***H. influenzae* SapA**

	Cluster 1	Cluster 2	Cluster 3	Cluster 4*
HADDOCK score	-145.2±3.3	-133.1±3.5	-129.0±5.1	-112.0±22.1
Cluster size	57	43	20	4
RMSD (Å)	0.5±0.3	0.6±0.1	0.7±0.0	0.6±0.0
Van der Waals energy	-30.6±2.5	-44.8±2.1	-44.8±6.5	-28.6±5.6
Electrostatic energy	-250.1±14.1	-116.5±42.4	-122.8±61.2	-163.8±131.8
Desolvation energy	-71.8±4.1	-67.8±6.9	-64.7±7.0	-60.6±5.0
Restraints violation energy	71.9±38.2	28.8±16.0	50.7±14.6	99.3±37.7
Buried surface area (Å^2)	984.3±68.8	1136.1±15.7	1115.1±52.7	887.9±96.5
Z-score	-1.7	-1.2	-1.0	-0.2

***H. influenzae* SapA with truncated loop**

	Cluster 1	Cluster 2	Cluster 3	Cluster 4
HADDOCK score	-136.1±5.0	-120.8±6.3	-115.2±7.0	-86.5±5.1
Cluster size	120	11	37	8
RMSD (Å)	0.4±0.2	0.6±0.0	0.5±0.0	0.5±0.0
Van der Waals energy	-32.0±8.6	-39.7±6.2	-142.8±13.9	-26.9±6.3
Electrostatic energy	224.7±46.5	-134.7±36.2	-51.9±10.4	-22.6±11.6
Desolvation energy	-65.8±5.6	-57.1±6.4	-51.9±10.4	-57.6±8.2
Restraints violation energy	65.6±38.1	28.8±16.1	13.3±20.0	24.5±24.4
Buried surface area (Å^2)	951.4±173.4	1089.7±86.4	1104.9±53	769.5±148.5
Z-score	-1.2	-0.3	0.0	1.6

***H. influenzae* PE**

	Cluster 1	Cluster 2
HADDOCK score	-66±1.0	-43.4±10.5
Cluster size	189	6
RMSD (Å)	1.1±0.2	1.1±0.1
Van der Waals energy	-40.8±5.2	-36.8±4.1
Electrostatic energy	-63.3±15.2	-46.6±18.5
Desolvation energy	-13.8±7.2	1.3±6.3
Restraints violation energy	11.8±11.0	14.9±11.0
Buried surface area (Å^2)	1147.0±72.4	1077.1±45.3
Z-score	-1.0	1.0

***H. influenzae* HxuA**

	Cluster 1	Cluster 2	Cluster 3	Cluster 4
HADDOCK score	40.2±3.4	-35.0±7.1	-13.6±10.7	-2.8±17.2
Cluster size	138	35	8	4
RMSD (Å)	0.4±0.3	0.5±0.1	0.4±0.0	0.5±0.0
Van der Waals energy	-40.0±4.7	-40.6±5.1	-21.5±4.7	-25.2±9.7
Electrostatic energy	-202.6±23.5	-111.3±41.0	188.1±55.6	-104.8±7.8
Desolvation energy	37.9±3.1	27.0±5.8	39.6±1.4	40.4±13.2
Restraints violation energy	24.3±18.2	8.5±4.2	59.6±17.5	29.4±13.4
Buried surface area (Å^2)	923.5±15.5	1021-2±45.7	671.6±66.3	723.5±92.2
Z-score	-1.4	-1.1	0.0	0.6

137

Table S5. Minimal inhibitory concentration of 16 antibiotics against NTHi WT and mutant strains determined by microdilution.

**Strain**	**AMP**	**AMC**	**CXM**	**FEP**	**CTX**	**CRO**	**IPM**	**MEM**	**CLO**	**TET**	**CIP**	**SXT**	**RIF**	**NAL**	**MXF**	**LVX**
NTHi375WT	0.25	<0,5/0,25	2	<0,25	<0.06	<0.12	1	0.25	<1	<1	<0.03	<0.05/9.5	<0.025	<4	<0.5	<0.5
NTHi375Δ*hxuCBA*	0.25	<0,5/0,25	1	<0,25	<0.06	<0.12	0.5	0.25	<1	<1	<0.03	<0.05/9.5	<0.025	<4	<0.5	<0.5
NTHi375Δ*hpe*	0.25	<0,5/0,25	2	<0,25	<0.06	<0.12	1	0.25	<1	<1	<0.03	<0.05/9.5	<0.025	<4	<0.5	<0.5
NTHi375Δ*sapA*	<0.12	<0,5/0,25	0.5	<0,25	<0.06	<0.12	0.12	0.25	<1	<1	<0.03	<0.05/9.5	<0.025	<4	<0.5	<0.5
NTHi375Δ*hbpA*	0.25	<0,5/0,25	0.5	<0,25	<0.06	<0.12	1	0.25	<1	<1	<0.03	<0.05/9.5	<0.025	<4	<0.5	<0.5

AMP: Ampicillin, AMC: Amoxicilin-Clavulanic, CXM: Cefuroxime, FEP: Cefepime, CTX: Cefotaxime, CRO: Ceftriaxone, IPM: Imipenem, MEM: Meropenem, CLO: Chloramphenicol, TET: Tetracycline, CIP:Ciprofloxacin, SXT: Cotrimoxazole, RIF: Rifampicin, NAL: nalidixic acid, MXF: moxifloxacin, LVX: levofloxacin

CLSI criteria for microdilution assay breakpoints (µg/ml): AMP: S (≤ 1), I (2), R (≥ 4); AMC: S (≤ 4/2), R (≥8/4); CXM: S (≤ 4), I (8), R (≥ 16); FEP: S (≤ 2); CTX: S (≤ 2); CRO: S (≤ 2); IPM: S (≤ 4); MEM: S (≤ 0.5); CLO: S (≤ 2), I (4), R (≥ 8); TET: S (≤ 2), I (4), R (≥ 8); CIP: S (≤ 1); SXT: S (≤ 0.5/9.5), I (1/19-2/38), R (≥ 4/76); RIF: S (≤ 1), I (2), R (≥ 4); MXF: S (≤ 1); LVX: S (≤ 2). S: Sensitive, I: Intermediate, R: Resistant.

References

1. Mason KM, Munson RS, Jr., Bakaletz LO. A mutation in the *sap* operon attenuates survival of nontypeable *Haemophilus influenzae* in a chinchilla model of otitis media. Infect Immun. 2005 Jan;73(1):599-608. doi: 10.1128/IAI.73.1.599-608.2005. PubMed PMID: 15618200.
2. Tanaka KJ, Pinkett HW. Oligopeptide-binding protein from nontypeable *Haemophilus influenzae* has ligand-specific sites to accommodate peptides and heme in the binding pocket. J Biol Chem. 2019 Jan 18;294(3):1070-1082. doi: 10.1074/jbc.RA118.004479. PubMed PMID: 30455346.
3. Notredame C, Higgins DG, Heringa J. T-Coffee: A novel method for fast and accurate multiple sequence alignment. J Mol Biol. 2000 Sep 8;302(1):205-17. doi: 10.1006/jmbi.2000.4042. PubMed PMID: 10964570.
4. Robert X, Gouet P. Deciphering key features in protein structures with the new ENDscript server. Nucleic Acids Res. 2014 Jul;42(Web Server issue):W320-4. doi: 10.1093/nar/gku316. PubMed PMID: 24753421.
5. Allen S, Zaleski A, Johnston JW, et al. Novel sialic acid transporter of *Haemophilus influenzae*. Infect Immun. 2005 Sep;73(9):5291-300. doi: 10.1128/IAI.73.9.5291-5300.2005. PubMed PMID: 16113244.
6. Tracy E, Ye F, Baker BD, et al. Construction of non-polar mutants in *Haemophilus influenzae* using FLP recombinase technology. BMC Mol Biol. 2008 Nov 11;9:101. doi: 10.1186/1471-2199-9-101. PubMed PMID: 19014437.
7. Rodriguez-Arce I, Marti S, Euba B, et al. Inactivation of the thymidylate synthase thyA in non-typeable *Haemophilus influenzae* modulates antibiotic resistance and has a strong impact on its interplay with the host airways. Front Cell Infect Microbiol. 2017;7:266. doi: 10.3389/fcimb.2017.00266. PubMed PMID: 28676846.
8. Whitby PW, VanWagoner TM, Seale TW, et al. Comparison of transcription of the *Haemophilus influenzae* iron/heme modulon genes *in vitro* and *in vivo* in the chinchilla middle ear. BMC Genomics. 2013 Dec 27;14:925. doi: 10.1186/1471-2164-14-925. PubMed PMID: 24373462.
